# Model-Based Scale-up Methodologies for Pharmaceutical Granulation

**DOI:** 10.3390/pharmaceutics12050453

**Published:** 2020-05-14

**Authors:** Eun Ha Jang, Yun Sang Park, Min-Soo Kim, Du Hyung Choi

**Affiliations:** 1 Department of Pharmaceutical Engineering, Inje University, Gyeongnam 621749, Korea; galaxy03313746@gmail.com; 2 College of Pharmacy, Chungbuk National University, Cheongju 28160, Korea; bbabbak22@naver.com; 3 College of Pharmacy, Pusan National University, Busandaehak-ro 63 heon-gil, Geumjeong-gu, Busan 46241, Korea

**Keywords:** scale-up, pharmaceutical granulation, engineering-based modeling, PAT-based modeling, physics-based modeling

## Abstract

In the pharmaceutical industry, it is a major challenge to maintain consistent quality of drug products when the batch scale of a process is changed from a laboratory scale to a pilot or commercial scale. Generally, a pharmaceutical manufacturing process involves various unit operations, such as blending, granulation, milling, tableting and coating and the process parameters of a unit operation have significant effects on the quality of the drug product. Depending on the change in batch scale, various process parameters should be strategically controlled to ensure consistent quality attributes of a drug product. In particular, the granulation may be significantly influenced by scale variation as a result of changes in various process parameters and equipment geometry. In this study, model-based scale-up methodologies for pharmaceutical granulation are presented, along with data from various related reports. The first is an engineering-based modeling method that uses dimensionless numbers based on process similarity. The second is a process analytical technology-based modeling method that maintains the desired quality attributes through flexible adjustment of process parameters by monitoring the quality attributes of process products in real time. The third is a physics-based modeling method that involves a process simulation that understands and predicts drug quality through calculation of the behavior of the process using physics related to the process. The applications of these three scale-up methods are summarized according to granulation mechanisms, such as wet granulation and dry granulation. This review shows that these model-based scale-up methodologies provide a systematic process strategy that can ensure the quality of drug products in the pharmaceutical industry.

## 1. Introduction

The granulation process is a commonly used process in the pharmaceutical industry. Through the granulation process, the shape and size of granules can be made large and uniform to increase the flowability and compressibility of powders [1,2]. Granulation processes are divided into wet granulation and dry granulation processes, depending on the use of binding solvent. Wet granulation is performed by spraying a binding solvent while agitating the powder. Granules are formed through capillary and viscous forces and permanent bonding between particle and binding solvent is completed by drying [3]. The process of wet granulation consists of three complex stages: wetting and nucleation; consolidation and growth; and breakage and attrition [4]. It is important to understand the effects of process parameters to gain insight into the complex process of granulation. The process parameters of wet granulation processes may vary depending on the process principle, such as high (low) shear granulation, fluidized bed granulation and twin-screw granulation. Generally, process parameters related to the force exerted on the granules (such as impeller speed and chopper speed) and the binders (such as binder amount, binder fluid viscosity and spray mode) can have a major impact on the quality attributes (QAs) of granules [5,6,7,8]. Granule quality can be measured by attributes, such as particle size distribution, granule hardness, bulk density, tapped density, uniformity, moisture content and flowability [9,10]. Unlike wet granulation, dry granulation is the process of agglomerating particles under pressure without the addition of a binding solvent [11]. Most dry granulation processes are performed using roller compactors [12,13]. Roller compaction is useful for the granulation of formulations containing heat- or moisture-sensitive active pharmaceutical ingredients (APIs) [14,15]. The roller compaction process consists of a feeding step employing a screw, a compression step employing two rolls and a step for crushing into granules [15,16]. Relative density, porosity and tensile strength are major QAs for ribbons, which are an intermediate product of dry granulation. The gap between the rollers and the forces, size and shape of the rollers are process parameters that can affect the QA of ribbons [17,18,19].

Early in the drug product development cycle, the formulation and process are developed using APIs and pharmaceutical excipients to ensure the quality, safety and efficacy of drug products at the laboratory scale [20]. The developed process can then be scaled up to larger batch sizes to reach the commercial scale. During the technology transfer stage from laboratory scale to commercial scale, the formulation developed at the laboratory scale is generally fixed; however, the process parameters are changed. For example, as the size of the container used in the granulation process increases, the volume or weight of the powder used in the process is increased and the impeller size, chopper size and range of operating speed can also be changed [21,22]. These changes can lead to changes in product quality. Successful scale-up can be achieved if the process parameters are appropriately adjusted to achieve the same quality as that observed on a laboratory scale [23].

In the past, researchers have performed a lot of troubleshooting to achieve the scale-up a process owing to the lack of fundamental process understanding and the absence of insights into unit operations. Unplanned troubleshooting is not only unscientific but also a waste of time and resources. To evolve beyond these past practices, a broad understanding of the pharmaceutical manufacturing process is needed. A successful scale-up requires a fundamental process understanding and insights into unit operations; these are based on mechanical insights into the process [24]. Simultaneously, the Food and Drug Administration (FDA) has introduced a Quality-by-Design (QbD) approach as a way to efficiently and quickly produce high-quality pharmaceutical products [25]. The scale-up of a manufacturing process should proceed in a manner that ensures the quality of the product in accordance with the QbD approach defined by the International Conference on Harmonization guidelines (ICH Q8: pharmaceutical development) [26]. To comply with these regulations, the ways in which variability is reduced during scale-up should be established based on a systematic understanding of the manufacturing process and using the QbD approach.

This review focuses on the application of three methodologies employed in pharmaceutical granulation processes. These three methodologies are the engineering-based modeling method, the process analytical technology-based (PAT-based) modeling method and the physics-based modeling method. A description of the basic theories and tools for these three methodologies is also provided. In addition, previous research into process control using these three methodologies is summarized in terms of wet granulation and dry granulation.

## 2. Engineering-Based Modeling Method

In the pharmaceutical scale-up step, the range of process parameters and the instrument geometry may be changed depending on the batch size, which may have a significant effect on the QAs. To minimize this effect, the pharmaceutical industry should use the same instrument series, such as a Collette Gral mixer (Gral 10–Gral 300), Fielder PMA mixer (PMA 10–PMA 1800), Diosna mixer (P10–P1250) or Powrex mixer (VG-1–VG-3000). Although, the geometrical similarity is secured in the scale-up step, the kinematic similarity and dynamic similarity should be carefully considered to obtain the required target drug quality profiles.

An engineering-based modeling method has been introduced to determine a scale-up strategy, which is based on process similarities among different scales [27]. The engineering-based modeling method describes functional relationships for defining process characteristics based on a dimensional analysis. A dimension is a qualitative description of a physical property, such as length or mass. The assumption for dimensional analysis is that the mathematical formulation of a physical process is valid in a system of any dimensions [10]. Dimensional analysis can reduce the number of experimental variables needed to correlate physical phenomena and thus produce dimensionless numbers to describe a process [27]. The dimensionless numbers are relevant to and identical for process states at all scales. The engineering-based modeling method considers different scale processes to be completely similar, such as in terms of geometrical, kinematic and dynamic similarity. Geometrical similarity represents the same linear scale ratio, such as in terms of the vessel dimension ratio and the impeller diameter. Kinematic similarity indicates that two systems have the same length to scale and time to scale ratios. For example, the impeller tip speed is controlled by the impeller rotational speed and blade diameter. Dynamic similarity indicates the same ratio of forces between corresponding points of two systems, which indicate similarities in internal particle flow, such as particle velocity and collision energy, during the granulation process [28].

The typical dimensionless numbers derived from dimensional analysis that can be used for mechanical device operation, heat transfer and mass transfer are shown in Table 1 [29]. Mechanical unit operation involves phenomena, such as material growth, material movement and material distribution. Heat transfer includes phenomena, such as evaporation and heat exchange. Mass transfer includes phenomena, such as gas absorption, distillation, extraction, adsorption and drying. Unfortunately, these dimensionless numbers cannot always achieve results in certain manufacturing environments. Additional complements may be required, such as the way different dimensionless numbers are used together [29].

Newton, Froude and Reynolds are the most commonly used methods to describe the scale-up process of granulation. The Newton number can quantitatively describe the trigger powder against friction in the fluid flow of materials in a granulation vessel. The number can be calculated with the torque or power consumption of the impeller in the granulation instrument. The Froude number can quantitatively describe the force pushing the materials against the granulation instrument wall. The Reynolds number is related to the viscous force, so it can be used to determine dynamic similitude between viscous flow at different scales [10].

## 3. PAT-Based Modeling Method

The PAT-based model method focuses on the monitoring of QAs in the process. Using the PAT-based model method, process parameters can be controlled in real time to obtain the target QAs, which do not depend on the batch scale of a process, the instrument geometry and the material properties [5,30]. Therefore, PAT can increase operational efficiencies, capacity utilization and process understanding [31]. A traditional quality analysis method may be more time-consuming and introduce various systematic errors [32]. Product quality can be analyzed in real time in processes using PAT as it has already been introduced in the pharmaceutical industry. In addition, it may present insights into the process mechanism to improve drug product quality [33]. Therefore, the following PAT case study is not limited to researches related to scale-up and includes studies confirming quality characteristics using PAT.

**Figure 1:**
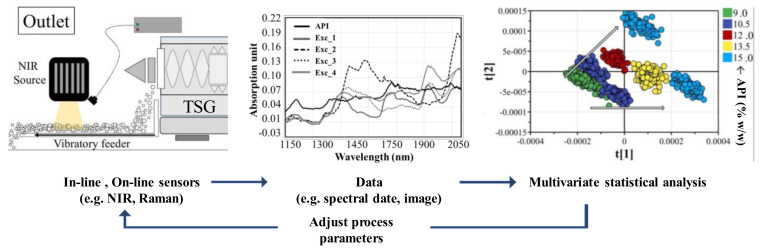
Summary of the approach to control the pharmaceutical manufacturing process using the process analytical technology tool [34]. The figure was modified, with permission from Elsevier.

The general sequence of controlling a process using PAT in a twin-screw granulator is shown in Figure 1. Three sensors (two sensors located at the entrance of the granulator and another sensor located at the outlet of the granulator) were used to monitor the API concentration. The spectral datasets obtained from the three sensors were evaluated using a multivariate analysis (MVA) model. Based on the MVA model, the API concentration was found to be significantly related to the change in powder density. Therefore, the API concentration was determined by monitoring the powder density [34]. This showed that the PAT-based modeling method is a useful tool to increase insight into the process and process scale-up.

Currently, various PAT tools have been introduced to analyze pharmaceutical processes in real time. One of the most commonly used PAT tools is near-infrared spectroscopy (NIR); NIR (electromagnetic waves in the region of approximately 800–2500 nm) can be used to analyze the molecular structure of materials depending on absorption in the NIR region. It can be used for the identification of materials or for the measurement of moisture content, density and particle size in various processes [35]. Raman spectroscopy is also used to distinguish materials by the detection of scattered light by determining the energy-absorption level difference of a sample molecule upon irradiation with monochromatic light with a wavelength of 532–1064 nm [36]. Raman spectroscopy can be used to identify the homogeneity of mixtures, granules and tablets [37,38].

**Figure 2:**
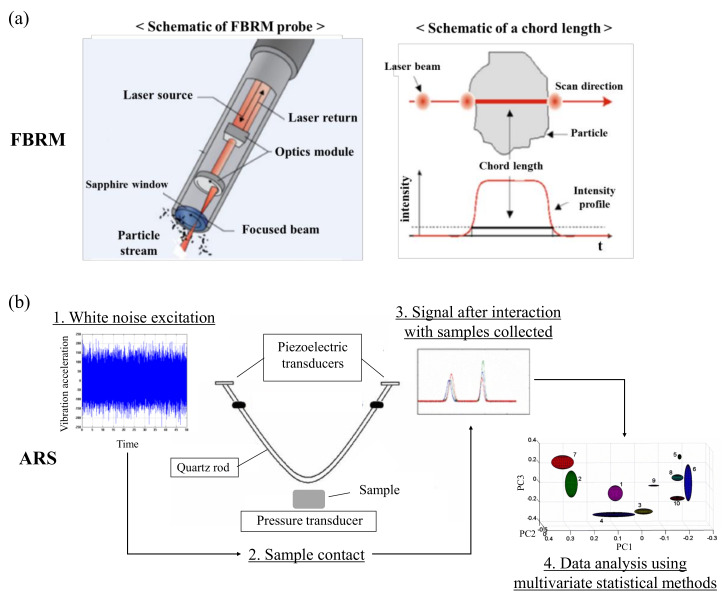
(**a**) Measuring principle of focused beam reflectance measurement (FBRM) [62]; (**b**) measuring principle of acoustic-resonance spectrometry (ARS) [63]. Slightly modified, with permission from Elsevier and Springer, respectively.

An imaging method in combination with an optical fiber and camera device can be employed to monitor particle size changes in real time. The imaging method measures particle size using a charge-coupled device camera and an optical fiber for illumination [39,40]. Focused beam reflectance measurement (FBRM) is a non-destructive way to characterize particle size using scattered light as the laser beam intersects the particle [41,42]. FBRM technology employs a probe-based instrument that is inserted directly into a container being used for a process to identify changes in particle size and count, particle structure and droplets in real time at full process concentrations. As shown in Figure 2a, the probe angle is controlled to ensure particles can flow easily across the probe window, where the measurement takes place. A laser beam is launched down the probe tube through a set of optics and is tightly focused onto a beam spot in the sapphire window. The optics rotate at a fixed speed, allowing the beam spot to rapidly scan particles as they flow past the window. As the focused beam scans across the particle system, individual particles or particle structures backscatter the laser light to the detector. These distinct pulses of backscattered light are detected and counted and the duration of each pulse is multiplied by the scan speed to calculate the distance across each particle. This distance is defined as the chord length, a fundamental measurement of a particle that is related to the particle size. Typically, thousands of particles are counted and measured per second, allowing the precise and highly sensitive chord length distribution to be reported in real time. The chord length distribution tracks how particle size and count change from the beginning to the end of a process. Trends in statistics for each chord length distribution, such as those regarding counts in fine and coarse size classes, can be observed over time. These particle size measurement methods have been introduced to determine the endpoint of granulation in real time [43].

Another image method is to use parsum^TM^ probe based on the spatial filter velocity principle. The parsum^TM^ probe can convert the laser light obscuration signal of individual particles into size information for analysis by the extended spatial filter as the particles pass through the hole at the probe tip. The measurable range is from 50 μm up to 6 mm [44]. The internal compressed air supply system can be used to keep sapphire windows clean. Both FBRM and parsum^TM^ probe can detect the moving particles with a laser beam. FBRM uses back scattered light and parsum^TM^ probe uses the generated shadow [45]. In addition, Eyecon^TM^ particle sizing technology is a 3D-imaging system to measure the particle size using a flash imaging technique. A strong light pulse is generated at the measurement, capturing a clear image of the particles. Illuminated by red, green and blue LEDs from multiple angles of the particle, the color of the particle surface is captured. The diameter can be obtained using surface color data [46].

Acoustic-resonance spectroscopy (ARS) is a non-destructive method to obtain seismic waves following the release of accumulated elastic energy after a material is deformed [47,48]. The wide applicability of ARS includes the determination of sample compaction and axial strain, deformation, hydration and drying endpoint, elasticity, molecular stacking and homogeneity [49]. In addition, ARS can rapidly and efficiently allow the identification and quantitative evaluation of samples [50]. As shown in Figure 2b, white noise from the transmitter is entered into the receiver through the quartz rod. When the sample contacts the piezoelectric transducers, AR spectrum was interfered with by sample and represent the particular spectrum pattern. Collected signals are analyzed statistical methods like multivariate method. ARS has been successfully used to monitor tableting processes [51,52], powder granulation processes [15,53,54] and semisolid and liquid blending processes [55,56].

Recently, water and density monitoring methods using microwave resonance technology has been reported for PAT. Materials containing water can absorb and reflect some microwave energy, allowing the measurement of their moisture content [57,58,59]. X-ray technology for PAT has also been reported. X-ray micro-computer tomography (X-ray micro-CT) is a non-destructive technology that can provide microscopic three-dimensional images at a micron resolution (from one to a few hundred microns), which is required for microstructural characterization [60,61].

## 4. Physics-Based Modeling Method

To use the engineering-based modeling method for scale-up, the geometric, kinematic and dynamic similarities for material movement in the process should be established. However, it is difficult to analyze similarities in the pharmaceutical process experimentally [64]. Currently, the material movement in the process is analyzed using physics-based modeling method. Physics-based modeling method can calculate the motion of materials using computational mathematical models and can display it as computer graphics. The use of physics-based modeling method is increasing with the development of computer technology. The physics-based modeling methods include Discrete element method (DEM), Computational fluid dynamics (CFD) and Finite Element Method (FEM). Additionally, the prediction ability can be improved by combining two or more of these models or combining one of them with a population balance model (PBM). One of the main advantages of physics-based modeling method in the scale-up step may be unrestricted predictability. This type of modeling can predict the result of manufacturing process regardless of batch scale or instrument geometry. In addition, it can present insights into processes, such as in terms of material collision force, material flow rate, number of intact bonds and number of materials, that cannot be obtained by experimentation. Although, physics-based modeling method has various advantages in the pharmaceutical process with the growth of computational technology, the challenging aspects of the physics-based modeling method are the reduction of practical run times and the need for a method using a polyhedral shape of material [65]. In particular, the computational cost and time of DEM simulation might be major constraints. In particular, in the DEM simulation on a commercial scale process, the calculation is limited as the number of particles increases. This is due to the high computational demands of the CPU (central processing unit) for the calculation of contact between particles and explicit time integration schemes [66]. The cost and time of computational can be reduced by combining various physics-based models.

### 4.1. DEM

**Figure 3:**
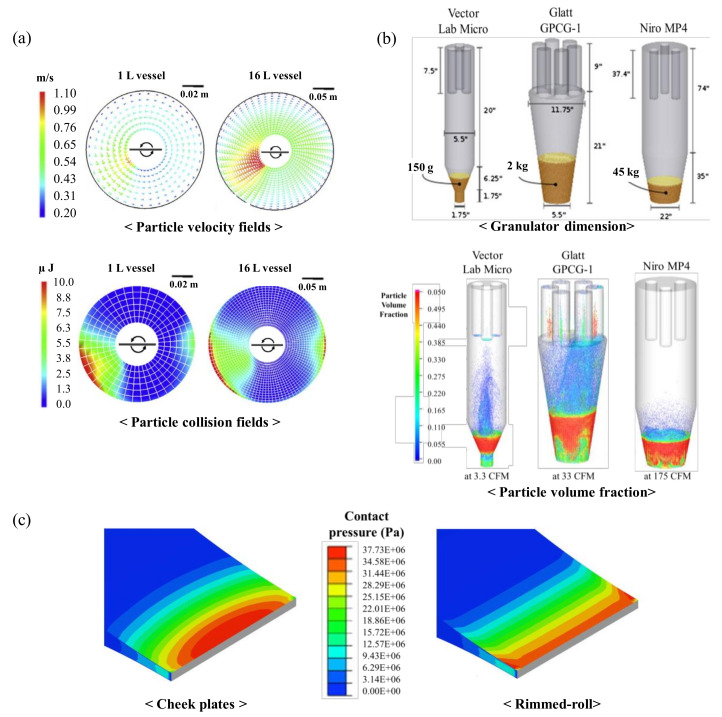
Application of physics-based modeling method for pharmaceutical scale-up process: (**a**) Discrete element method (DEM) predictions (particle velocity and collision fields) in a high-shear granulator (1 L and 16 L) [64]; (**b**) Computational fluid dynamics (CFD) simulation in three scale of fluidized bed granulator [78]; (**c**) FEM simulation for compared contact pressure with cheek plates and rimmed-roll [79]. The figures were slightly modified, with permission from Elsevier.

DEM is used to simulate mechanical dynamics, such as particle position, particle velocity and motion of individual particles, which can predict pharmaceutical processes, such as powder blending, die filling, hopper discharge and twin-screw feeding and conveying. For each particle, Newton’s law is used to predict particle motion [67,68,69]. The commercial software programs for DEM are Chute Analyst (Overland Conveyor Co., Inc.), EDEM^TM^ (DEM Solutions Ltd.), PFC^TM^ (Itasca consulting group, Inc.), SimPARTIX^®^ (Fraunhofer IWM) and Rocky DEM^TM^. The open-source software for DEM are Mercury-DPM [70], YADE [71], LIGGHTS [72] and MFIX-DEM [73]. To calculate the particle position using DEM, the force generated due to the collision of individual particles with the wall is calculated using a contact model. The calculated force is then used to calculate the velocity of each particle to determine the new position of the particle. This procedure is repeated during the time step and the particle position can be recalculated continuously [74]. DEM allows the analysis of particle behavior and energy acting on particles, which is not dependent on the batch scale, instrument geometry or material properties used in a process. In addition, DEM can reveal the dynamics of and insights into the system and provide numerical values that might be hard to obtain experimentally. As shown in Figure 3a, the particle velocity and the particle collision energy can be obtained in-progress. To identify particle movements in 1 L and 16 L high-shear granulators, the particle velocities and the particle collision energies were compared. The particle collision energy of different batch sizes, which are dynamically similar, was similar under normalized agitation power. However, particle velocities, which are kinematically similar, differed depending on the batch size. This might indicate that DEM is an effective tool to identify the kinematic and dynamic similarities of granulators in the scale-up step [64].

### 4.2. CFD

CFD is a process design and analysis tool, which can simulate the behaviors of a fluid, such as fluid flow and heat flow. The basic principle of CFD is to separate the solution domain into a number of control quantities and integrate these control quantities into algebraic equivalents to transform partial differential equations. The solution of these equations by numerical technology has paved the way for relatively easy flow field calculations [75]. Well-calibrated CFD simulations can provide detailed insight into the physics of a flow system and make non-invasive flow, heat and concentration field predictions [76]. ANSYS^®^, ANSYS Fluent (Ansys Inc.), Flow-3D^®^ (Flow science, Inc.), COMSOL Multiphysics^®^ (COMSOL Inc.) and Autodesk CFD (ALGOR Inc.) are commercially available CFD software. Also, OpenFoam^®^, an open source for CFD simulation, is available [77]. In the pharmaceutical granulation process, CFD can be applied to simulate the flow of air in fluidized bed granulation. As shown in Figure 3b, three different fluidized bed granulators (Vector Lab Micro, Glatt GPCG-1 and Niro MP4) were simulated using CFD. The flow of particles in the fluidized bed granulation process are presented by the color of particles, according to the particle volume fraction. CFD has been used to predict the fluidized bed granulation operating results that are dependent on process parameters, such as inlet air flow rate, fluidized bed size and shape and material properties, that represent the scale-up step of fluidized bed granulation [78].

### 4.3. FEM

The process simulation using FEM is divided into finite elements or volumes. The powder behavior in the process is calculated from partial differential equations that conserve mass, momentum and energy [65]. FEM can show the pharmaceutical process results, such as dry granulation and tableting, of powder behaviors in the presence of high pressure [23,79,80]. As shown in Figure 3c, the predictive results, such as the force exerted for ribbons and the relative density of ribbons on the roller compaction process, can be presented graphically. The results showed that there was a significant wall effect between the roller surface and the ribbon. The contact pressure was dependent on the part of the roll and different contact pressures in different parts of a roll may result in different relative densities on the ribbon. These results may be difficult to obtain experimentally; thus, FEM can provide greater insight and understanding of the roll compaction process [79]. The ANSA Pre-processor (BETA CAE System), FreeFem++, ANSYS^®^ (Ansys Inc.), CalculiX [81] and other similar commercial and open source software can be used to determine the dynamics of compaction.

### 4.4. Coupled Physics-Based Modeling

The pharmaceutical manufacturing process is a complex association of many phenomena and it is sometimes simulated by coupled models [82]. Models coupled with PBM are often used to investigate particle size changes, particle growth rate and particle dynamics in the granulation process [83,84,85]. The collision energy and velocity of the particles can be predicted using DEM and the data obtained are used to calculate the particle size from the aggregation and breakage kernels of PBM. The coupled modeling method has been used for pharmaceutical milling processes [36] and pharmaceutical granulation processes [84,86]. A DEM–CFD coupled modeling method can be used to predict particle behaviors in fluid systems, such as the fluidized bed granulation process. DEM can calculate the position and velocity of individual particles and CFD provides a profile of the fluid flow at each time step [87,88]. In addition, coupled DEM–CFD-PBM modeling is used to simulate the fluidized bed granulation process [82,89]. The effects of operating parameters, such as inlet volumetric air flow rates, inlet air temperatures and superficial gas velocity, on particulate flow dynamics can be estimated using a two-way coupled CFD and DEM model. PBM can be used to calculate the rate of particle aggregation and breakage and predict the particle size with particle dynamic information from the CFD–DEM simulations. This showed that the framework of the coupled multi-phase model can be used to understand fluid-particle dynamics in fluidized bed granulation in the scale-up step [82].

## 5. Scale-Up Strategy for Wet Granulation

In wet granulation, granules are formed by the addition of a granulation liquid onto a powder bed that is under the influence of an impeller (in a high-shear granulator), screws (in a twin-screw granulator) or air (in a fluidized bed granulator) [5]. The resulting agitation in the system, along with the wetting of the components within the formulation, results in the aggregation of the primary powder particles to produce wet granules. The binding solution contains binders, such as povidone, polyvinyl pyrrolidone (PVP) and hydroxypropyl cellulose, which can form bonds between powder particles that are strong enough to lock them together and thereby minimize the potential for segregation and poor content uniformity. Wet granulation can produce more spherical and uniform and larger particles to increase compressibility and enhance powder flowability [28,29].

For successful wet granulation process development and scale-up, a researcher should have comprehensive knowledge of the principle of operation and identify the effect of process parameters on drug product quality and the QAs of the wet granulation process. As shown Table 2, wet granulation can be divided into three main processes, including high (or low) shear, twin-screw and fluidized bed granulation, according to the equipment type. The process parameters are dependent on the type of equipment. In addition, the critical properties of the process parameters may be determined by risk assessment and experimental design [90]. Although the critical process parameters may differ depending on the type of equipment and formulation developed, the QAs of granules may be similar after the wet granulation process. The content and content uniformity of API in granules are significantly related to the content and content uniformity of the final product. Granule size, granule size distribution, bulk density and tapped density may be significantly related to granule flowability, which could have effects on various processes, such as die filling, hopper discharge and twin-screw feeding and conveying. It is important to control the process parameters and maintain the QAs of granules upon scale-up step of wet granulation [91].

### 5.1. Scale-Up Studies on Wet Granulation Using an Engineering-Based Modeling Method

**Figure 4:**
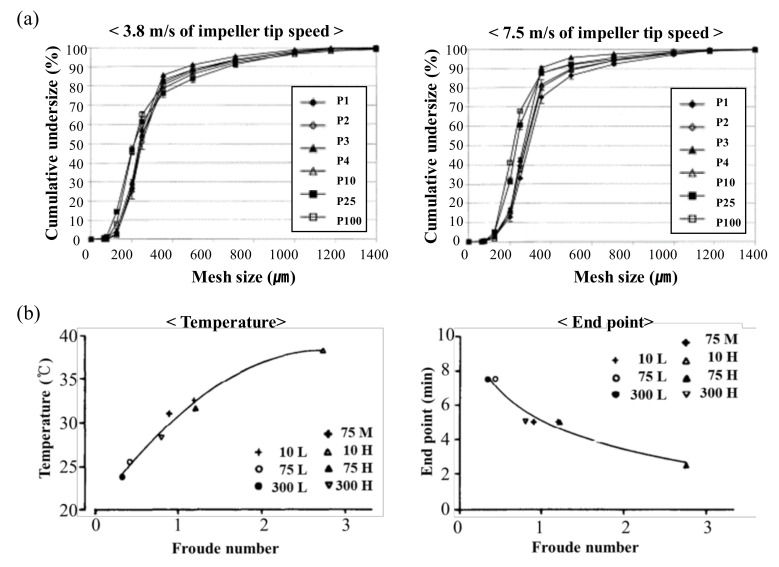
Scale-up studies using the engineering-based modeling method: (**a**) particle size distribution according to bowl size (P1–P100) to maintain the same impeller tip speed: 3.8 and 7.5 m/s [99]; (**b**) relationship of Froude number with temperature and end point [100]. The figures were slightly modified, with permission from Elsevier.

The engineering-based modeling method for the scale-up of the wet granulation process is presented in Table 3 by equipment type. The scale-up conditions, critical process parameters controlled in the scale-up step, measured QAs of granules and dimensionless numbers are presented from the relevant studies. The most commonly used engineering-based modeling method can be sorted into impeller speed-related [9,92], power consumption-related [93,94,95] and binder-related [96,97] models. The three common dimensionless numbers associated with impeller speed in the abovementioned models are constant impeller tip speed, relative swept volume and Froude number, respectively. The impeller tip speed can be expressed as follows:

(1) Impeller tip speed=πnd,

where *n* is the centrifugal speed in rotation/min and *d* is the impeller diameter. Rekhi et al. studied the scale-up of a wet granulation process using 10, 65, 150 and 300 L Fielder granulators (GEA Niro Aeromatic Fielder, Paris, France). In their study, impeller tip speeds were maintained constantly for approximately 5 and 10 m/sec using four vessel sizes (10, 65, 150 and 300 L). Granule QAs, such as particle size distribution, moisture content, bulk density, tap density and content uniformity, were similar for all four vessel sizes. The impeller-rotating speed was decreased with an increase of impeller diameter to maintain impeller tip speed [98]. Moreover, Bock et al. studied scale-up using the Diosna granulator (Diosna Dierks und Söhne GmbH, Osnabrück, Germany), with a constant impeller tip speed. The impeller speed was controlled according to the vessel size to maintain the same impeller tip speed: 3.8 and 7.5 m/s. This study suggested that even if the impeller tip velocity was the same, the particle size distribution may vary depending on the bowl size. As shown in Figure 4a, the granule size distributions prepared in Diosna P1–P10 had similar profiles [99].

Froude number is a commonly used dimensionless number associated with the impeller speed in wet granulation. Froude number was calculated using the following equation:

(2) $$Froude\;number=\frac{rpm^{2}D}{g},$$

where rpm is revolutions per min, *D* is the diameter of the impeller and *g* is the gravitational constant. Froude numbers represent the ratio between the centrifugal force and the gravitational force. They may indicate the dynamic similarity between granulators. Horsthuis et al. compared the mixing behavior using lactose in three sizes (10, 75 and 300 L) of Collette Gral (GEA-Colette, Wommelgem, Belgium) to identify scale-up parameters. The temperature and size distribution of the granules were evaluated as QAs. The end time point was determined when a change in particle size no longer occurred. The relationship between the Froude number, the endpoint and the temperature is shown in Figure 4b. Equal Froude numbers result in similar processes with respect to temperature and particle size distribution. A constant relative swept volume and a constant impeller tip speed may result in a different process [100].

Another scale-up parameter associated with the impeller is the relative swept volume. This dimensionless number is defined as the volume of granules swept per unit time by the impeller or blade divided by the total volume of the granules. Depending on the shape of the impeller and the blade used, the calculation method is changed. Holm et al. investigated the impeller and chopper design effect on granule growth by swept volume using a Fielder PMAT 25 laboratory high-shear mixer. Their results indicated that the greater the amount swept, the higher the density and the narrower the granule size distribution [101]. Similar to the relative swept volume, a dimensionless number is used, which is defined as the agitation force divided by the mass of the whole granule [102,103,104]. Sato et al. measured the agitator torque and then calculated the agitator torque per unit vessel volume. In this study, the agitator torque per unit vessel volume was strongly correlated with the physical properties of granules, such as size, strength and compressibility. This may show that unit vessel volumetric agitation force can be applied as a scale-up parameter [104].

In addition, a scale-up study using power consumption was conducted. Power consumption is often used in combination with other dimensionless numbers and parameters. Some researchers [8,105] have scaled up a process using a number of dimensionless correlations related to power consumption. Landin et al. investigated a fixed bowl mixer-granulator scale-up using Power, Reynolds and Froude numbers. Different sizes of granulators were geometrically similar. The powder bed height variation was applied as a scaling factor correcting the dimensionless number. It can be applied to predict the granule end point for 25,100 and 600 L machines [105]. Faure et al. proposed a dimensionless number relative to power consumption. Regarding the fill level, the relationship between the Power number, the Reynolds number and the Froude number was established. In addition, experiments were conducted at different blade speeds in planetary mixers of different sizes to produce similar wet mass characteristics. When wet mass characteristics (size distribution, density and flow) were similar, the QAs of dry granules were similar [8].

Dimensionless numbers relating to the spraying of binders have been proposed. Litster et al. presented a granulation dimensionless number of scale-up as the proportion of sprayed binder liquid and flux value of the powder bed. The flux value was calculated using the following equation [97]:

(3) $$\Psi =\frac{3V}{2Ad},$$

where *A* is the flux value of the powder surface traversing the spray zone, *V* is the volumetric spray flow rate and *d* is the average drop size. A dimensionless spray flux can be used to determine how dense the binder is on the powder surface. Increasing the spray flux causes the binder to overlap and form larger granules. To form granules of the same quality at any scale, the dimensionless spray flux must be kept constant. Ax et al. investigated the effect of the spray droplet size of the binder on the nucleation step of the granulation process using a spray flux created using the intensive mixer, Eirich R02 (EIRICH Machines, Inc., Hardheim, Germany). Different nozzles types (0.3, 0.5 and 1.2 mm aperture diameter) were used to change the size of the droplets and the resulting granules were analyzed for size and moisture content. A correlation was found between droplet size and granule size. The initial particle size was different according to the droplet size. However, when increased the time of break and reaggregation steps in the granulation process, the effect of droplet size was ignored. In this study, it described that particle size can be affected by droplet size, but, 5 min after the addition of the binder, mechanical stresses dominated than the droplet size [106].

### 5.2. Scale-Up Studies on Wet Granulation Using a PAT-Based Modeling Method

The PAT-based modeling method for the scale-up of the wet granulation process is presented in Table 4 by the equipment type. The used PAT tools, critical process parameters controlled in the scale-up step and measured QAs of granules are presented from the relevant studies.

The method for monitoring the growth of granules in wet granulation is the measurement of the power consumption relative to the wet mass properties. Power consumption is considered the first PAT tool to monitor high-shear wet granulation [116,117]. Watano et al. measured and analyzed power consumption as a monitoring method for wet granulation using a high-speed mixer. Power spectral specific peaks obtained by fast Fourier transform analysis of power consumption was used to evaluate the quantitative relation between the change in the intensity of the specific peak and the granule growth [118]. It is possible to monitor the power consumption curve and compare the different batch sizes of the granulation process but the power consumption is widely used with dimensionless relationships [110]

NIR is a most commonly used PAT tool for monitoring the wet granulation scale-up step. In addition, it can measure the QAs of granules, such as moisture content and content uniformity and can provide information in a non-destructive and quick manner [119]. Frake et al. studied the real-time monitoring of moisture content using NIR in a fluidized bed granulation scale-up step. The moisture content of granules may be a significant determinant of the endpoint of fluidized bed granulation, regardless of batch size. Spectra between 1100 and 2500 nm were collected during the process and the peaks that corresponded to the OH bonds in the water were isolated. The moisture content was determined based on NIR spectra, which indicated the endpoint of fluidized bed granulation. To validate the endpoint obtained through NIR spectra monitoring, loss on drying and Karl Fisher titration were performed. The validation result showed that in-process NIR monitoring of water content was suitable for process control and endpoint determination for a fluidized bed granulation scale-up step [120].

**Figure 5:**
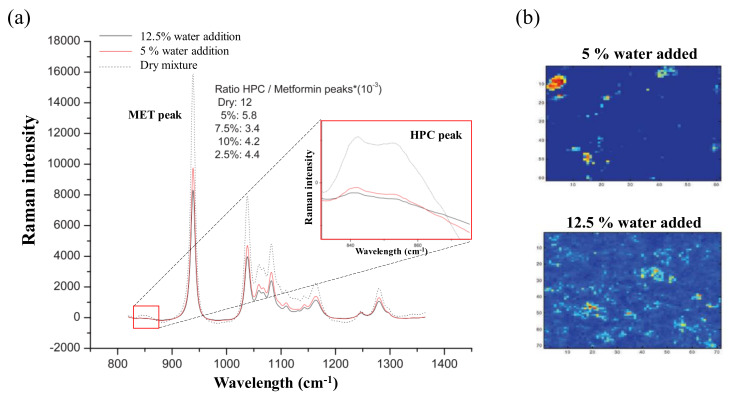
(**a**) Ratio between hydroxypropyl cellulose (HPC) peak (850 cm^−1^) and metformin peak (938 cm^−1^) in granules with 5% and 12.5% water and the dry mixture. Strong magnification of the peak at 850 cm^−1^ (inset graph) shows the intensity of the HPC band; (**b**) Raman intensity mapping image [38]. The figures were slightly modified, with permission from SAGE journals.

Raman chemical mapping can also be used to identify content uniformity in wet granulation processes. Content uniformity is an important QA to monitor the success of wet granulation process scale-up. Sasic et al. studied the change and distribution of intragranular binders in the MiPro granulator (ProCepT, Zelzate, Belgium) using Raman spectroscopy. The formulation containing metformin, hydroxypropyl cellulose (HPC) and microcrystalline cellulose (MCC) was used to identify the HPC Raman band in the range of 820–1390 nm upon the addition of water. As shown in Figure 5a, HPC was found to respond strongly to the addition of water, with its domain dissipating and Raman bands weakening as water was added. This suggests that Raman chemical mapping has different intensities in the presence of water, such as in wet granulation processes. As shown in Figure 5b, mapping images presented that the intensity of hot pixels was decreased when the water content increased. Based on the monitoring, Raman spectroscopy was used to observe the QAs of granules during the wet granulation scale-up step [38]. A similar study was conducted by Wikström et al., who used Diosna granulator (Diosna Dierks und Söhne GmbH, Osnabrück, Germany), an RXN1-785 Raman spectrometer and NIR-250L-1.7T2 NIR spectrometer to monitor the solvent-mediated pseudo-polymorphic conversion of the API. Wet granulation processes involve the addition of an aqueous binder. Unlike NIR, which can be disturbed by water, Raman spectroscopy can clearly display the scattering of the API, because water does not have a strong Raman spectrum [121].

**Figure 6:**
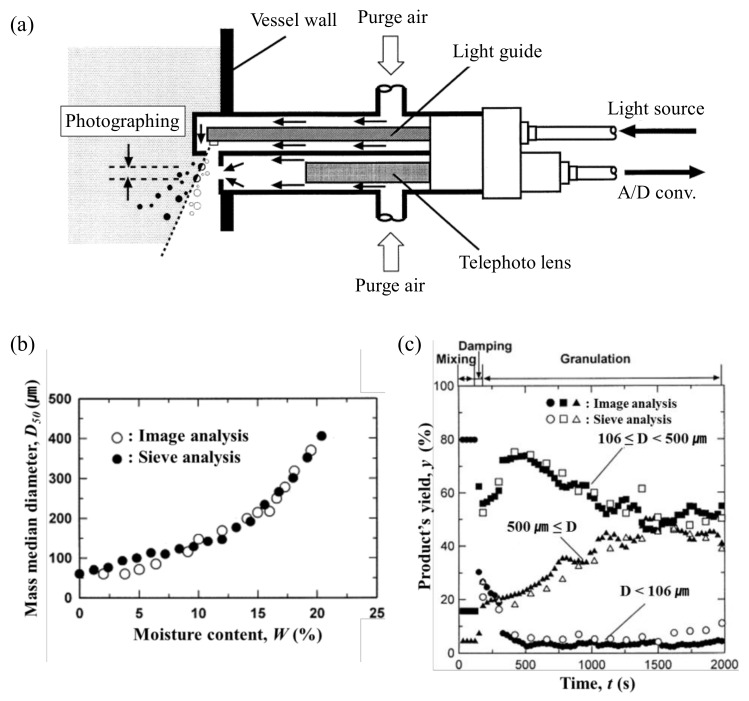
(**a**) Schematic diagram of image probe. A/D conv. is analog-to-digital converter; (**b**) the relationship between the moisture content and the median diameter of granules; (**c**) the results of yield for each granule size measured during the granulation process. Circles represent a particle size of less than 106 μm, squares represent particle sizes of 160–500 μm and triangles represent particle sizes of 500 μm or more [39]. The figures were slightly modified, with permission from Elsevier.

The techniques for measuring the real-time change in particle size in the granulation process include imaging technology and FBRM [41,122]. The particle size, particle size distribution or particle growth rate present evidence to determine the endpoint of wet granulation; thus, their QAs can be used for the wet granulation scale-up step. Watano et al. studied an image analysis method to determine the endpoint for laboratory-scale fluidized bed granulation (NQ-160 Fuji Paidal Co., Ltd., Osaka, Japan). Images were collected using a charge-coupled device camera with optical fibers for lighting as shown in Figure 6a. The high energy lamp provided flashing light at 1μs intervals. At the extremity of the probe, light up a thin plane at an angle through the slit. The charge-coupled device camera focuses on the plane, granules on the lit-up plane can be detected and their sizes measured correctly. The comparison of particle sizes determined through image analysis and sieve analysis was performed. Figure 6b shows the particle size change according to moisture content and Figure 6c shows the differences in product yield between the three particle sizes. The image analysis showed a good correlation for a real experiment using sieve analysis, which can contribute to the scale-up process development of granulation [39].

Tsujimoto monitored the fluidization phenomenon by measuring the amplitude of acoustic emission waves generated by the impact and friction between the granulator walls and particles during the fluidized bed granulation process. The fluidized bed granulator, AGM-2A-PJ (Hosokawa Micron Ltd., Osaka, Japan) was monitored using an acoustic emission sensor (AE-901S, NF Kairo Setukei Block) with a 140 kHz resonance point. The influence of the parameters (inlet air velocity, particle size, moisture content, rotating disk speed and bed hold-up) was investigated by measuring the amplitude of the average acoustic emission. It was confirmed that the acoustic emission measurement technique can indicate particle behavior in fluidized bed granulation; however, it has been found that as the fill level increases and the particle size density decreases, monitoring using acoustic emission propagation becomes more difficult [48]. 

### 5.3. Scale-Up Studies on Wet Granulation Using a Physics-Based Modeling Method

The physics-based modeling method used for the scale-up of wet granulation processes are presented in Table 5 by equipment type. The used simulation tools, critical process parameters controlled in the scale-up step and predicted QAs of granules are presented from the relevant studies.

**Figure 7:**
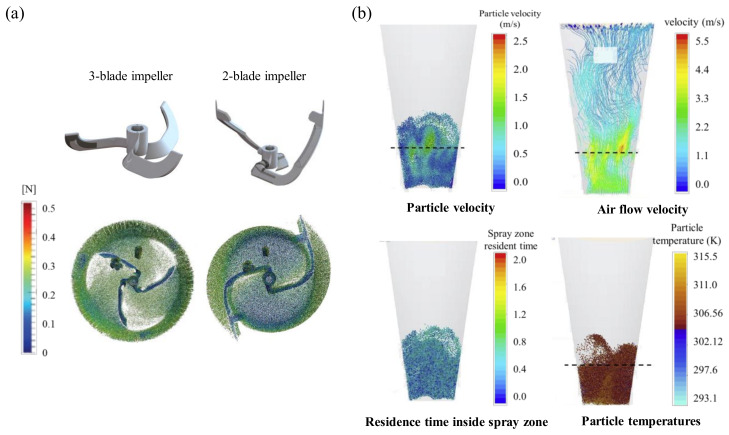
Scale-up process simulation using a physics-based modeling method: (**a**) shear force comparison according to the different blade type [6]; (**b**) CFD–DEM coupled simulation: particle velocity, airflow velocity, residence time inside and particle temperatures [82]. The figures were slightly modified, with permission from Elsevier.

A physics-based model can be used to compare and analyze the kinematic similarity of the granulation process. The simulation of the wet granulation process was initially conducted using PBM [131,132]. PBM calculated the change in particle number for each particle size class. This often requires information about flux data, impact frequency and impact force. DEM can be used to obtain the required information [84]. Börner et al. used DEM to compare the flow behaviors of granules in the high-shear granulation process depending on the number of blades and the shape of blades, as shown in Figure 7a. A high rotation speed should be provided to initiate powder circulation for three-blade impellers. With high rotation speeds, a huge amount of energy is introduced in the powder, which may induce undesirable thermal stress and increase granule breakage rate [6]. A new impeller design consisting of two blades was investigated to improve the mixing and granulation behavior. The mixing behavior was experimentally investigated in a laboratory mixer (10 L in volume) and at production scale (600 L). The mixing homogeneity of colored sugar pellets was investigated by digital image analysis (DIA) at several impeller rotation speeds. DEM simulations were used to obtain shear forces and force distributions on a single-particle scale. The mixing investigations showed that the two-blade impeller had great potential for scale-up. The DEM simulation indicated that both impeller types applied almost the same shear force on particles [6]. In addition, Chan et al. studied the effect of rotating impeller blade forces on granule properties, which are subsequently transmitted throughout by inter-granule collisions. The blade-granule bed normal stress in a cylindrical, vertical axis high-shear granulator was characterized using DEM simulations. The simulations were compared and validated by measuring the blade-bed stress and bed surface velocities [133].

CFD is used to simulate flow in a fluid and can be coupled with DEM to predict the interactions between fluids and particles in scale-up step using fluidized bed granulation [82]. Tamrakar et al. developed a combined CFD–DEM–PBM framework to investigate fluidized bed wet granulation. The simulation was performed using a GPCG fluidized bed dryer (Glatt Air Techniques Inc., Ramsey, NJ, USA) and a top-spray fluidized bed granulator. CFD was used to predict the flow of air in fluidized bed granulation, DEM was used to predict particle behavior in the fluidized bed granulator and PBM was used to calculate particle growth rate. Simulation can have predicted the attributes of particle velocity, airflow velocity, residence time inside spray zone and particle temperatures as shown in Figure 7b. Using the developed model, the effects of critical process parameters (such as inlet air rate, the temperature of the air and spray rate) of the fluidized bed granulator were evaluated to determine critical QAs, including granule size distribution and liquid content value [82]. This study suggests that coupled physics-based modeling (hybrid modeling) can be used to investigate the effect of process parameters on critical QAs resulting from the scale-up of fluidized bed granulation.

**Figure 8:**
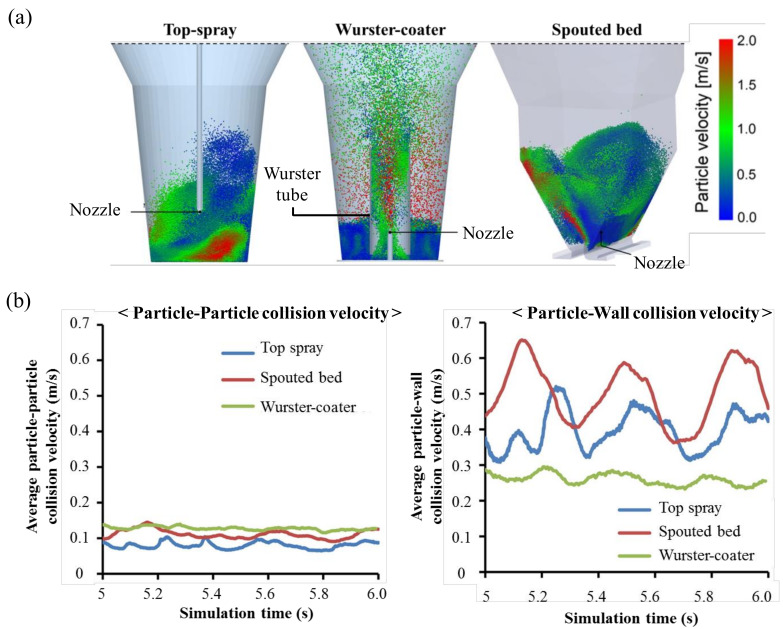
Comparison of three fluidized bed granulators (Top-spray, Wurster-coater and Spouted bed): (**a**) simulation snapshots under the same process conditions, particle flow and particle velocity fields were captured; (**b**) profiles of the particle-particle and particle-wall collision velocities within the stable fluidization regime (during a simulation time of 5–6 s) [134]. The figures were slightly modified, with permission from Elsevier.

Fries et al. used a coupled DEM–CFD framework to compare the three granulator configurations. Simulations were used to predict the mean particle velocity, the interparticle and particle-to-equipment collision rates and the collision frequency. Simulation snapshots of the particle flow and particle velocity fields of each granulator under the same process conditions are shown in Figure 8a. In addition, the average relative collision velocity was obtained over time, as shown in Figure 8b. The average relative particle-particle collision velocity was not significantly different between the granulators but the particle-wall collision speed was different. These results allow the calculation and comparison of the agglomeration probability, growth rate and cohesive strength of granules [134]. This study suggested that a coupled DEM–CFD method could predict the powder behavior for different geometries and process conditions at the scale-up step.

**Figure 9:**
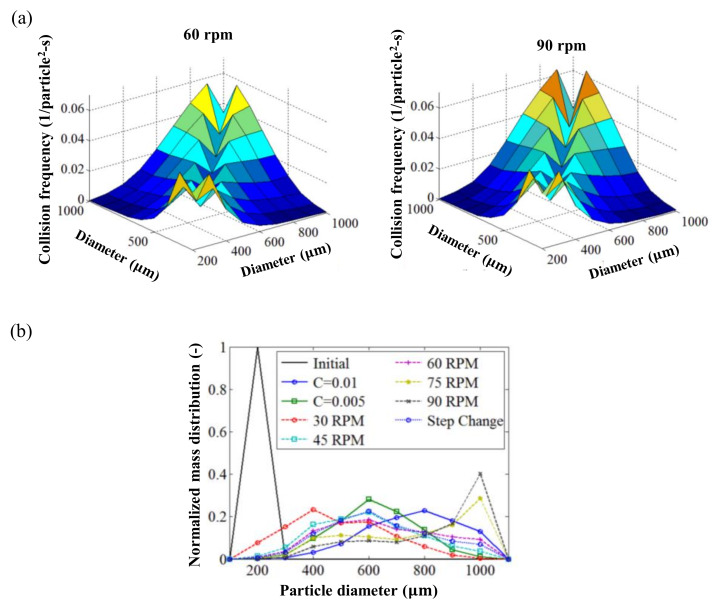
(**a**) Simulation result of collision frequency predictions at 60 and 90 rpm impeller speeds; (**b**) particle size distribution after 180 s depending on the constant collision frequencies (0.01 and 0.005) and impeller speeds (30–90 rpm) compared with the initial particle size distribution [84]. The figures were slightly modified, with permission from Elsevier.

In addition to a coupled CFD–DEM framework, a coupled DEM-PBM has been used to simulate the granulation process because the simulation of the particle growth phenomenon might be the most important QA for wet granulation. Barrasso et al. presented a coupled DEM-PBM framework to simulate wet granulation. PBM was used to predict changes in the number of particles in each size class due to rate processes, such as aggregation, which often rely on empirical rate kernels or require additional mechanistic information, such as flux data, collision frequencies and impact forces. DEM presented each particle individually and showed the ability to simulate spatial variations and collect mechanistic data. DEM was used to determine the collision rates between particles of various sizes and then PBM used these data to determine aggregation rates. The predicted collision frequencies obtained using DEM at different impeller speeds is shown in Figure 9a. PBM was able to calculate the aggregation rate and the amount of particles by size using the results from DEM. The particle size distribution obtained under each simulation condition is shown in Figure 9b. The developed model confirmed the sensitivity of particle size to the impeller speed [84]. This study suggests that a coupled DEM-PBM framework can be used in the scale-up step to identify the effect of process parameters, such as impeller speed, on the QAs of granules.

Dhenge et al. studied the particle surface velocity according to the powder feeding rate and the viscosity of the binder using DEM in a twin-screw granulator. Particle Image Velocimetry (PIV) and DEM simulation presented the surface velocities for the dry and the wet formulation powders. Granulation at both a high viscosity of binding solution and powder feed rate resulted in small granules, strong bonds between the particles and a high compressibility index of the granules. The PIV indicated that the dry formulation powder had a higher velocity compared to the that of wet granules. The surface velocity increased with an increase in the viscosity of the granulation liquid and decreased with an increase in the powder feed rate [135]. This study presents a qualitative understanding of the twin-screw granulation process, which can be used to control the twin-screw granulation process in scale-up steps.

## 6. Scale-Up Strategy for Dry Granulation

**Figure 10:**
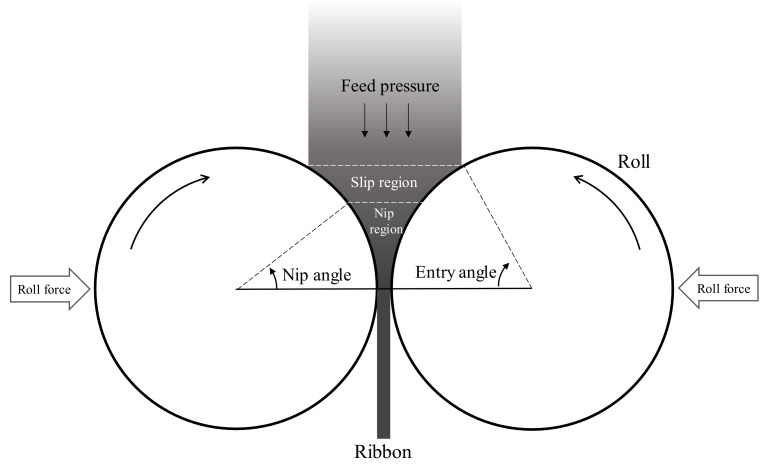
Schematic of the roller compaction process.

Dry granulation is applied widely in the manufacturing of pharmaceutical products. Roller compaction is the most commonly used dry granulation method. Dry granulation offers many advantages over wet granulation, including easier process control and cost benefits. In the wet granulation process, liquid is added to agglomerate the powder; a drying process is thus later required. This step is not required for dry granulation processes, making them simpler and more suitable for heat- or water- sensitive APIs [142]. Although the principle and operation of the roller compaction process are simple, a thorough understanding of the mechanism of roller compaction is still lacking [143]. The schematic of the roller compaction process is shown in Figure 10. The scale-up of the roller compaction study was conducted using traditional large-scale experimental designs to determine the optimization process for the design space. This was time and resource intensive [16,142]. To achieve a successful scale-up of roller compaction processes with only a small number of experiments, it is necessary to understand the process parameters and attributes of the ribbons and granules, which are the process products [17]. The main process parameters that need to be adjusted in the roller compactor are roll spacing, roll pressure, feed screw speed, roll speed and roller shape. The product of roller compaction, the ribbon, can be evaluated for attributes, such as density, strength, thickness, Young’s modulus, shape and moisture content.

### 6.1. Scale-Up Studies on Dry Granulation Using an Engineering-Based Modeling Method

In many published scale-up studies, process parameter settings were predicted using a dimensionless number to achieve a target ribbon density value. Maintaining ribbon density at different scales is important, because similar ribbon densities exhibit similar particle properties. However, scale-up in roller compaction is difficult because the nip shape and size do not increase proportionally as the roll diameter increases [142]. Pietsch proposed the following relationship between the diameter of the roller, the compressive force and the compressive pressure in the process of increasing the manufacturing scale of a roller compactor [144].

(4) $$\sqrt{\frac{Roll\;diameter_{2}}{Roll\;diameter_{1}}}=\frac{Compaction\;pressure_{2}}{Compaction\;pressure_{1}}\;or\;\frac{Compaction\;force_{2}}{Compaction\;force_{1}}.$$

Sheskey et al. scaled up roller compaction using the following simple equation. The scale-up was from laboratory scale to pilot and full scales and the model drug was theophylline, a controlled release agent. Granules made using the full-scale process through this method tended to produce a finer powder and dissolve faster than the laboratory-scale product [145].

(5) $$\frac{\mathrm{Roll}\;\mathrm{speed}}{\mathrm{Screw}\;\mathrm{speed}}=\mathrm{constant}\;\frac{\mathrm{Force}}{\mathrm{Linear}\;\mathrm{inch}\;\mathrm{of}\;\mathrm{roll}}=\mathrm{constant}.$$

**Figure 11:**
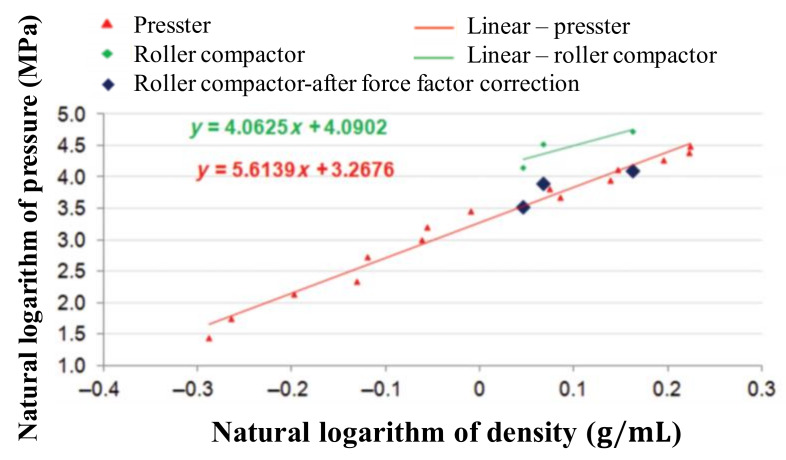
Plot of density against pressure applied using a presser and roller compactor [147]. The figure was slightly modified, with permission from Elsevier.

The dimensionless number frequently used in roller compaction is determined based on the Johanson theory [146]. Johanson proposed a model that predicts the density of ribbons made by roller compaction using the nip area and the volume between the roll gaps and further attempts have been made to refine Johanson’s theory. Bi et al. studied Johanson’s rolling theory using a machine with three model formulations. Inadequate assumptions made using Johanson’s model in previous studies resulted in inaccurate predictions of the maximum roll surface pressure and were corrected using mass correction factors in the study. The density *versus* predicted maximum roll surface pressure is shown in Figure 11. This shows that ribbons made from roller compactors require higher pressure than pressers to produce compacts of the same density. The modified Johanson’s rolling theory suggested that the model could be accurately predicted when compared with ribbon density but that it should be recognized that this model has limitations as a one-dimensional model [147]. To improve the accuracy of Johanson’s model, which used a fixed nip angle, Nasarikar et al. developed a modified Johanson’s model that excluded pressure from the nip angle. The model developed for WP 120 Pharma (Alexanderwerk, Remscheid, Germany) predicted ribbon density that compared reasonably with actual experimental values. Furthermore, the researchers analyzed if it could be scaled up using a WP200 roller compactor. The ribbon density of the model matched that of the batch prepared in WP200 with experimental data, indicating the success of the scale-up procedure [14].

**Figure 12:**
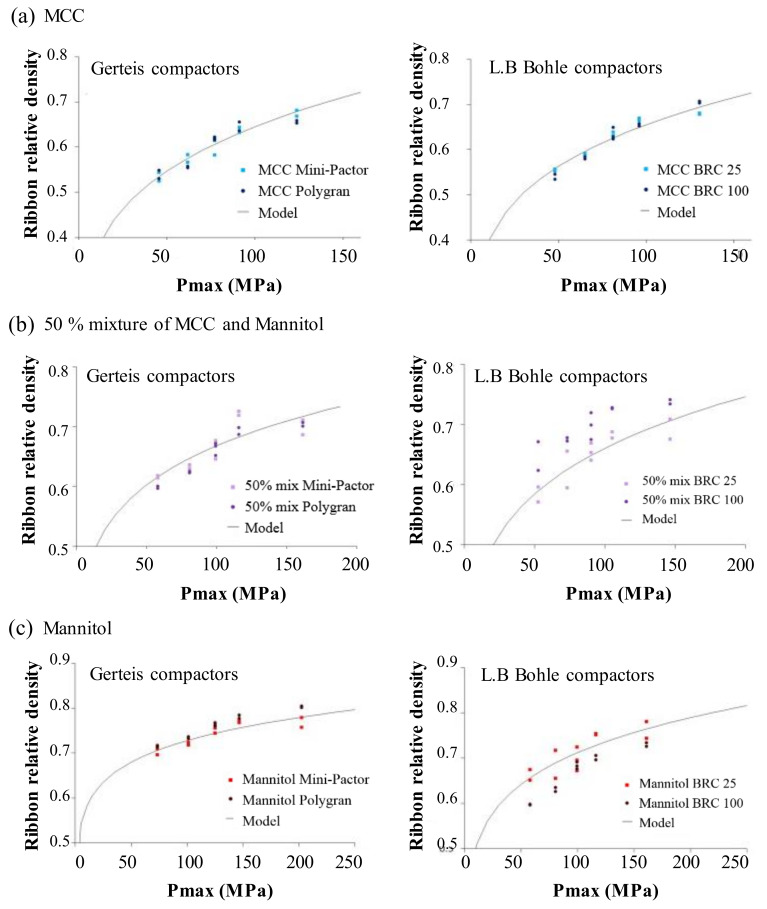
Comparison of the relative ribbon density of model predictions and experimental results on two roller compactors (Gerteis and L.B. Bohle compactors) [17]: the formulations used were (**a**) microcrystalline cellulose (MCC); (**b**) a 50% mixture of MCC and mannitol; and (**c**) mannitol. The figures were slightly modified, with permission from Elsevier.

As a means to eliminate the need for nip pressure, Reynolds et al. presented the effects of roller process parameters (roll force and roll separation), equipment geometry (roll diameter and roll width) and material properties (internal friction angle, wall friction angle and compressibility) through dimensionless numbers related to ribbon density [148]. This method included the feed screw speed and could be used to scale-up using the same geometry, as well as to change to other types of roller compactors. Gago et al. used a Gerteis compactor (Gerteis, Rapperswil-Jona, Swiss) and an L.B. Bohle (L.B. BOHLE, Ennigerloh, Germany) machine to determine the effect of scale-up using Reynolds’ proposed scale-up rule. The materials used were microcrystalline cellulose (MCC) and mannitol. The design of experiments using factors, such as specific compressive forces, gap widths and roll speeds, was obtained and characterized using the relative density of the resulting ribbons. As shown in Figure 12a, simulations using the MCC formulation showed fewer errors and good predictions. For a 50% mixture (Figure 12b) and mannitol (Figure 12c), the predictability of the model was reduced but the predictions were still within the 4.1% maximum error limit. The developed model predicted the performance of the Gerteis compressor better than that of L.B. Bohle machine [17].

Toson et al. divided the operation mode of the roller compaction into a gap-controlled mode and a screw-controlled mode and this study suggested that the Johanson’s model is more suitable for the gap-controlled mode and Reynold’s model is more suitable for the screw-controlled mode. However, these models overestimated the ribbon density. To correct the ribbon density, a calibration routine using compressibility factor and pre-consolidation density was introduced. Through calibration, material amount required and the number of roller compaction experiments can be reduced. The proposed method was able to predict the design space for a broad range of throughputs with high accuracy with a small number of experiments [149].

Rowe et al. extended Johanson’s model and proposed a modified Bingham number (Bm*) that represented the ratio of yield point to yield stress as follows:

(6) $$Bm^{*}=\frac{C_{S}}{\gamma_{0}\rho_{true}\pi D^{2}W}\frac{{\left(SA_{roll}\right)}^{0.5}}{S}\frac{N_{S}}{N_{R}}$$

where $C_{S}$ is the screw speed constant, $\gamma_{0}$ is preconsolidation factor, $\rho_{true}$ is true density, π is circumference of the roll circle, *D* is the roll diameter, *W* is roll width, $SA_{roll}$ is roll surface area, S is roll gap, $N_{S}$ is feed screw speed and $N_{R}$ is roll speed. Bm* is easy to determine because the input parameters of Bm* consist of those that can be generally measured in the compaction process. The model-predicted values and the actual test results from WP 120 Pharma and WP 200 Pharma (Alexanderwerk, Remscheid, Germany) models are shown. By maintaining Bm*, it was possible to obtain a consistent ribbon density between the two operating scales. It was suggested that Bm* can be effectively used for the development of roller compaction scale-up [143]. Case studies suggest that dimensionless numbers for the prediction of ribbon density in dry granulation processes can be used successfully during the scale-up process. Studies that have used engineering-based modeling method for dry granulation processes are summarized in Table 6.

### 6.2. Scale-Up Studies on Dry Granulation Using a PAT-Based Modeling Method

As dry granulation using roller compaction is a continuous process, PAT can be used to maximize the benefits of the continuous process. Dry granulation PAT tools commonly used in roller compaction include NIR, microwave resonance, Raman spectroscopy and X-ray. Using these techniques, ribbon density, moisture content tensile strength, Young’s modulus and API concentration can be measured in real time [155].

**Figure 13:**
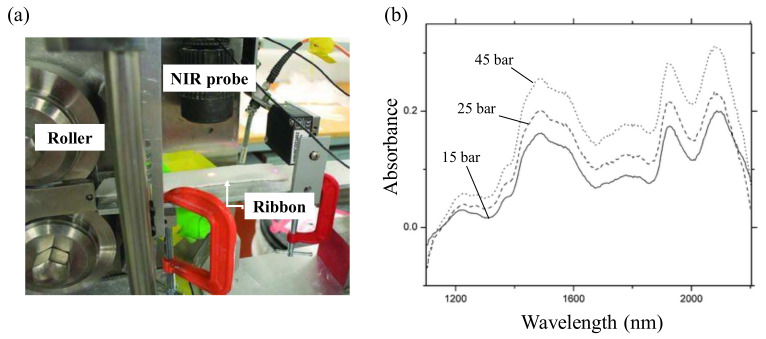
Near infrared (NIR) spectrum measurement of density made at various pressures (15, 25 and 45 bar) [156]: (**a**) in-line setup in roller compactor; (**b**) spectrum obtained at 1100–2205 nm. The figures were slightly modified, with permission from Springer.

In addition, NIR is useful in roller compaction to measure the real-time QAs of ribbons and granules, such as density, tensile strength, Young’s modulus and API concentration. Acevedo measured the ribbon density of a laboratory-scale roller compactor (Alexanderwerks, Remsheid, Germany) using a CDI non-contact diffuse reflectance spectrometer (SNIR 278, Control Development Inc. South Bend, IN). Figure 13a shows in-line setup in roller compactor. The slope of the spectrum obtained at 1121–1306 nm was used to measure the ribbon density, which is dependent on the pressure of the roll. As shown in Figure 13b, the spectra were obtained at different pressures. The correlation between the slope and the pressure of this spectrum shows a linear relationship. This study suggested that the MVA models can predict the ribbon density in a broad density range [156].

**Figure 14:**
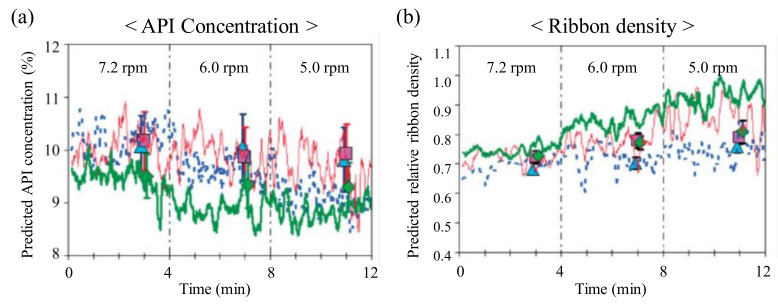
Predicted values estimated from NIR data at a relative humidity of 24% (blue), 45% (red) and 65% (green). Active pharmaceutical ingredient (API) concentrations determined using UV analysis are represented as triangles (24% RH), squares (45% RH) or diamonds (65% RH). The roll speed was changed every 4 min [157]: (**a**) API concentration (%); (**b**) relative ribbon density. The figures were slightly modified, with permission from Elsevier.

Gupta et al. proposed a model to predict ribbon QAs based on the slope of the best-fit line through the NIR spectra. Predictive models have also been developed in conjunction with Partial least squares (PLS) regression to predict ribbon density, moisture content, tensile strength and Young’s modulus. Ribbons were prepared at 24%, 45% and 65% relative humidities using a Chilsonator IR220 (The Fitzpatrick Co., IL, USA). NIR data were used to predict API concentration (Figure 14a) and relative ribbon density (Figure 14b). The roll speed was changed to 7.2, 6.0 and 5.0 every 4 min. As the roll speed decreased, the API concentration decreased and the ribbon density tended to increase [157].

**Figure 15:**
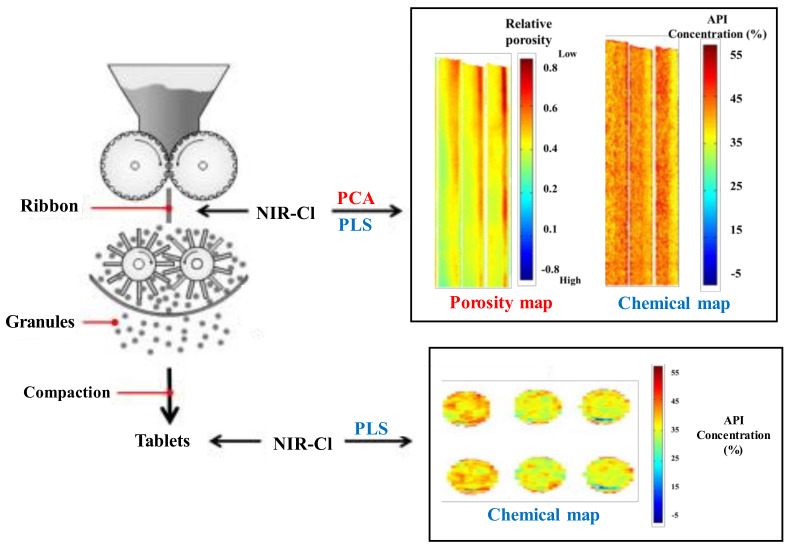
Summary of the use of NIR in roller compaction and tableting processes. Principal component analysis (PCA) and Partial least squares (PLS) were used to monitor the porosity and content uniformity of the ribbons and tablets, respectively [158]. The figure was slightly modified, with permission from Elsevier.

The introduction of near-infrared chemical imaging (NIR-CI) into the pharmaceutical process has been recently suggested. This technique can be used to gather information on ribbon density and API content. Khorasani et al. monitored the porosity and API content of a ribbon according to roll pressure and roll speed using NIR-Cl and presented a predictable model for granule size distribution. The overview of the application of NIR monitoring throughout the roll compression and tableting process is shown in Figure 15. PCA-based NIR-Cl was used to map ribbon porosity and PLS-based NIR-Cl was used to map the API distribution and content. Therefore, NIR-CI to monitor the quality characteristics of granules was established. This can be allowed processes of another scale to produce the same quality granules by monitoring ribbon density and API content [158].

**Figure 16:**
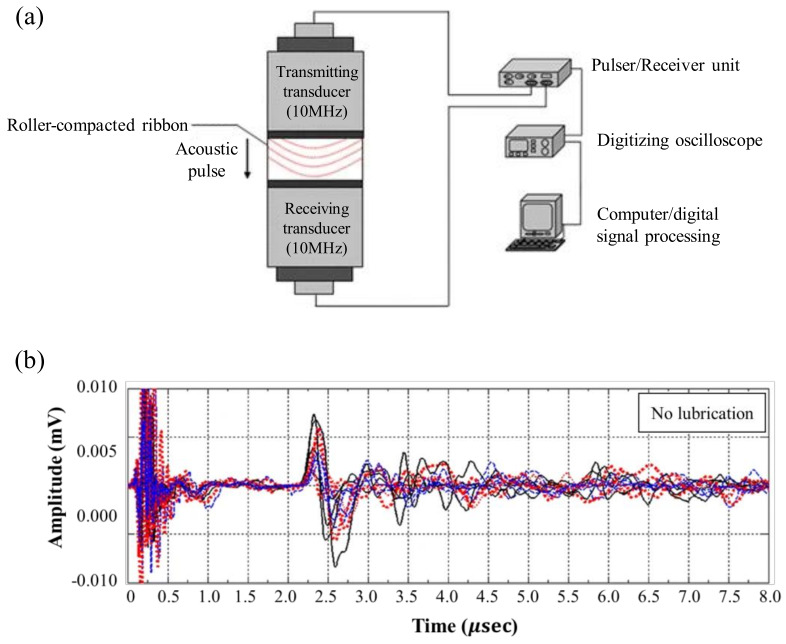
Application of non-destructive ultrasonic waves to predict ribbon characteristics [159]: (**a**) experimental setup; (**b**) acoustic waveforms obtained according to the position of the ribbon. Blue, black and red lines represent the scan at the upside, middle and the downside of ribbon, respectively. These results are for powder with no lubrication. The figures were slightly modified, with permission from Springer.

In addition, microwave resonance can be used as a PAT tool to measure the moisture content and density of the ribbon by recording the signal generated during powder compression and converting it into the frequency spectrum [57,58]. Microwave resonance has the advantages of requiring fewer calibration standards and higher penetration compared with NIR. Moisture-sensitive microwave resonance gives more accurate results for moisture measurement but does not provide chemical information [58]. The propagation of acoustic waves (wave packets) can be used to measure ribbon QAs, because it depends on the mechanical properties of the material (mass density, Young’s modulus and Poisson’s ratio). Akseli et al. quantified the tensile strength of a ribbon using an ultrasound spectrum. This non-destructive ultrasound method is based on the measurement of the flight time of the sonic pulse (ultrasound frequency bandwidth) passing through the specimen from the transmitting transducer (Panametrics, V129-RM) to the receiver unit (Panametrics, 5077PR), as shown in Figure 16a. The waveforms obtained for the specified ribbon locations in the study are shown in Figure 16b. In this study, through the measurement of acoustic waves, the QAs of the ribbon can be predicted and the properties of the granules can be evaluated. Despite variations in scale, through measurement of acoustic waves can be maintained consistent granule quality [159].

**Figure 17:**
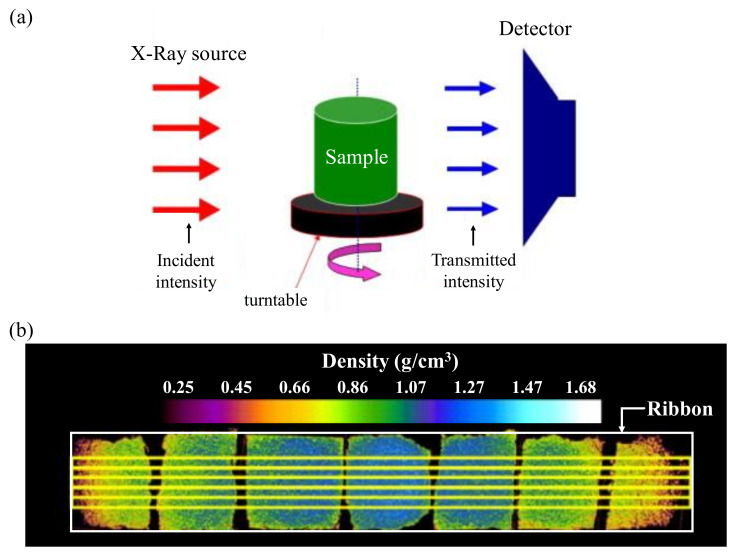
Photo-montage of integrated X-ray micro-CT images [160]: (**a**) measurement principle; (**b**) integrated micro-CT images of the ribbon samples. The figures were slightly modified, with permission from Elsevier.

Miguélez-Morán investigated the density distribution of ribbons in a roller compaction process using X-ray micro-CT. Tablets for simulation calibration were made using the same MCC as that used for making ribbons. X-ray micro-CT can be supplied three-dimensional images of micro-structures in non-destructive way. As shown in Figure 17a, sample is rotated and an X-ray beam is then directed to the sample. X-ray beam are absorbed when passing through the sample and others are transmit through the sample and be detected by the detector. Integrated micro-CT images for the prepared ribbon are shown in Figure 17b. This demonstrates that X-ray micro-CT can quantify changes in ribbon density [160].

More recently, new approaches have been developed to measure the density of ribbons. Wiedey et al. applied an infrared thermography camera to monitor the in-line ribbon density and visualize the density distribution within the ribbon. The ribbon temperature was correlated with the compression force and the relative density and the cooling rate after compaction could be used to predict the density more accurately. Infrared thermography has been proven with an inexpensive in-line measurement tool with high spatial resolution [161]

Many techniques for monitoring roller compactors can help understand the behavior of powder compaction in roller compactors. The studies that have applied PAT to dry granulation are summarized in Table 7.

### 6.3. Scale-Up Studies on Dry Granulation using a Physics-Based Modeling Method

Research into roller compaction using a physics-based modeling method is in progress to achieve a comprehensive understanding of the roller compactor process and successful scale-up. The structure of a typical roller compactor is divided into parts for screw feeding and parts compressed into two rollers. Although simple in construction, the mechanisms of the two parts are significantly different, making numerical modeling of the process difficult [23]. In the roller compaction process, DEM is suitable for simulating the movement of particulate solids in the feeding step and FEM is suitable for simulating the compaction step. In this section, the focus is on the simulation of the region being compressed by rolls, rather than the study of the effect of screw simulation on the flow of particles.

**Figure 18:**
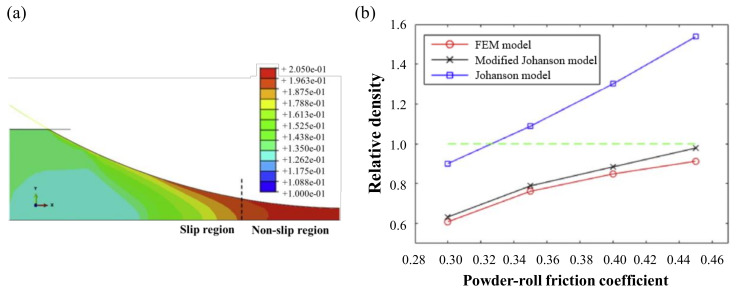
(**a**) Material velocity field using a finite element method (FEM) simulation, the dashed-line indicates the location of nip angle in Johanson’s model; (**b**) relative density as functions of powder-roll friction coefficient at three models; FEM, modified Johanson and Johanson model [18]. The figures were slightly modified, with permission from Elsevier.

Muliadi et al. predicted the nip angle, normal stress and maximum material relative density, according to the roll gap using FEM in the roller compaction process. The predicted values were compared with results obtained using Johanson’s model. The nip angles predicted in this study tended to be the same for Johanson’s model and FEM simulation. However, the compact density predicted using Johanson’s model was larger than that predicted using FEM. In addition, the normal stress predicted using Johanson’s model was more exaggerated than that predicted using FEM. To achieve more consistent simulations using the two approaches, the material should be more compressible and the internal friction angle, as well as the flow direction inlet vertical stress, should be low [165]. Liu et al. used FEM simulation to modify Johanson’s roll compression model to improve ribbon density predictability. Johanson’s model has been predicting a higher relative density for the powder because it assumes that the stream speed is equal to the roll speed. However, the powder velocity predicted by FEM was faster on the surface, as shown in Figure 18a. Therefore, they modified it to include fitting parameters that can be measured online using the existing Johanson’s model. The relative densities as functions of powder-roll friction coefficient is shown in Figure 18b. In the modified Johanson’s model, the mean relative density prediction was found to be approximately 5% greater with FEM. The modified Johanson’s model can be used for a more accurate estimation of the relative density of the ribbon [18].

**Figure 19:**
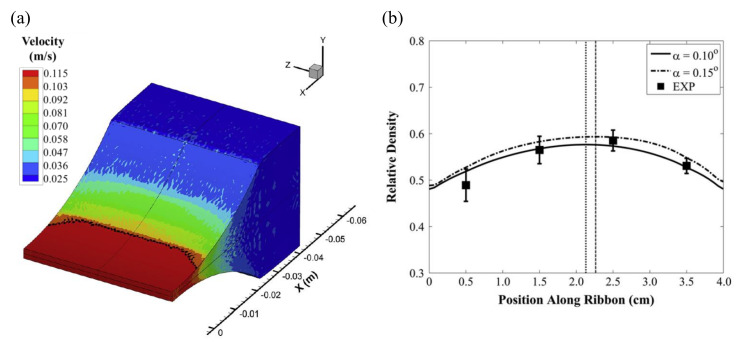
Predicted result by FEM simulation [166]: (**a**) powder velocity distribution at 0.12 m/s roll; (**b**) ribbon density distributions using inlet stress and roll gap were 164 kPa and 2.32 mm. α is roll tilt. The figures were slightly modified, with permission from Elsevier.

A study comparing the density results of actual experiments using the FEM model and the Alexanderwerk WP120 roll compactor (Alexanderwerk GmbH, Germany) was conducted. To improve the accuracy of the model, the boundary conditions of the model were determined using experimentally measured values. The powder velocity at 0.12 m/s roll velocity was observed through simulation, as shown in Figure 19a. The powder near the cheek-plate site experience a lot of friction, showing a slower speed than that of the powder in the center. The distribution of the ribbon density when the model inlet stress and roll gap were 164 kPa and 2.32 mm, respectively, is shown in Figure 19b. The actual roller compactor used was tilted at approximately 0.15°. To reflect this, the FEM simulation was performed using angles of 0.10° and 0.15°. The simulation results agree well with the experimentally observed density. This study suggested the potential of FEM for use in QbD.

**Figure 20:**
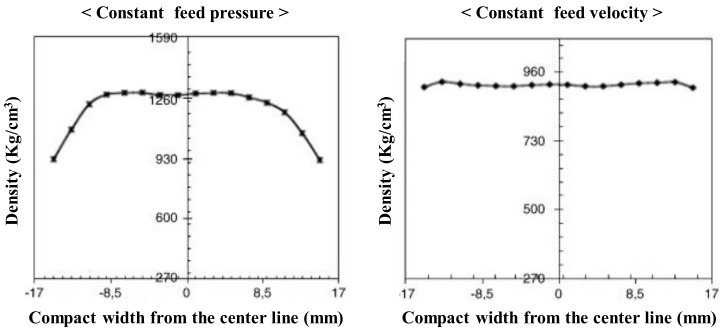
Roller compaction process simulation using FEM [167]: predicted density distribution along the width of the ribbon constant feed pressure (**left**) and feed velocity (**right**). The 0 on the x-axis means center in the width of the ribbon. The figures were slightly modified, with permission from Elsevier.

Moreover, a study on the effect of the process parameters of the roller compactor was conducted using FEM. Michrafy et al. used FEM to study how the inlet feed condition affected principal stress and density. The ribbon was produced by a Komarek B050PH laboratory press with a horizontal feed screw. The numerical simulation of roller compaction was performed using a commercial software (Abaqus explicit version 6.5). As shown in Figure 20, a constant supply pressure resulted in uniform maximum stresses and densities throughout the ribbon. However, simulations with constant feed rates showed the highest maximum principal stress at the center of the ribbon. The simulation was verified against mercury porosity, which is the data for the experiment [167].

**Figure 21:**
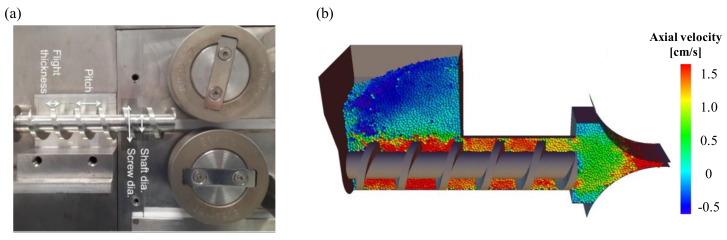
Komarek B050H roll compactor [23]: (**a**) set up of the experiment; (**b**) snapshot of simulation using DEM. The figures were slightly modified, with permission from Elsevier.

Combined three-dimensional DEM and FEM was developed by Mazor et al. to simultaneously simulate the feeding step and the compaction step of the roller compactor. Upon combining these two simulations, advantages of both simulations complement each other. Using the DEM simulation in the screw feeder region, the non-uniform velocity field of the particles between the feeder and the roller was successfully simulated. The actual and simulated roller compactors used are shown in Figure 21a. Furthermore, FEM was applied in the compaction area to simulate powder compaction according to non-uniform feed rate (Figure 21b). The non-uniform density distribution of the compressed ribbon was found to be due to the non-uniform feed rate. The DEM-FEM coupled model allows for more realistic simulations of the process and more accurate predictions [23].

Based on these studies, it is suggested that physics-based modeling method can improve insight into the mechanism of the roller compaction process. The studies that have used physics-based modeling method for dry granulation processes are summarized in Table 8.

## 7. Conclusions

Scale-up is the process of changing production from a laboratory scale to a pilot or commercial scale. Successful scale-up results in products with constant QAs, regardless of scale. However, it is difficult to achieve scale-up with just a few experiments. Therefore, a scientific approach is needed for successful scale-up to reduce the number of experiments and develop a fast and efficient scale-up strategy. In this review, three methodologies applicable to pharmaceutical granulation process scale-up was presented. In addition, the highlighted case studies show how each methodology was used in the actual pharmaceutical granulation processes. Engineering-based modelling method is a way to compare two processes by simply generating formulas that describe the process through dimensional analysis when the similarity between processes is assured. This method is relatively simple but there are many practical hurdles to its use in granulation scale-up steps and it may be hard to achieve similarity between processes. In wet granulation, dimensionless numbers related to the impeller speed are commonly used to maintain the kinematic and dynamic movements. PAT-based modeling method identify CQAs using a real-time monitoring tool. This tool can reduce the probability of process failure by adjusting process parameters in-process to achieve the desired quality and shortens the time required to measure QA so that an efficient process can be achieved. However, to apply the monitoring tool, an appropriate machine and a calibration step are required. Physics-based modeling methods calculate the motion of an object through a process-related physics equation and present it in graphical form. Physics-based models can be used to predict results in place of actual experiments or to gain insight into the process through results that cannot be obtained experimentally. In the pharmaceutical industry, DEM can simulate the behavior of particles, CFD can be applied for fluid simulation and FEM can simulate behaviors under pressure. The three methodologies for scale-up have been widely used in a variety of pharmaceutical granulation processes to achieve successful scale-up. A thorough understanding of the science-based methodology of scale-up should be viewed as a part of QbD approach, which requires scientific justification based on continuous improvement and knowledge management. This review can provide a suitable strategy and guidance to perform the scale-up of pharmaceutical granulation. In addition, this review presents the basis for further studies regarding the scale-up strategy in other pharmaceutical unit operation such as blending, compression, milling and coating and the future pharmaceutical industry such as continuous manufacturing with the goal of real-time release and a regulatory perspective for the QbD approach.

## Figures and Tables

**Table 1:** Commonly used dimensionless numbers related to machine operation, heat transfer and mass transfer.

Type	Name	Equation	Nomenclature
Mechanical unit operation	Reynolds, Re	νl/v	ν = velocity l = characteristic length v = liquid volume g = gravitational acceleration F = force ρ = density
	Froude, Fr	$\nu^{2}/lg$	
	Newton, Ne	$F/\rho \nu^{2}l^{2}$	
Heat transfer	Nusselt, Nu	hl/λ	h = convective heat transfer coefficient l = characteristic length λ = thermal conductivity t = time ρ = density $C_{p}$ = heat capacity at constant pressure
	Fourier, Fo	$\lambda t/\left({\rho C}_{p}\right)l^{2}$	
	Prandtl, Pr	$v\left({\rho C}_{p}\right)/\;\lambda$	
Mass transfer	Sherwood, Sh	kl/D	k = mass transfer coefficient l = characteristic length D = vessel diameter v = liquid volume ν = velocity $D_{ax}$ = axial dispersion coefficient
	Schmidt, Sc	v/D	
	Bodenstein, Bo	$\nu l/D_{ax}$	

**Table 2:** Process parameters and quality attributes (QAs) of granule by granulator type.

Type	Process Parameters	QAs of Granule
High-shear, Low-shear	Order of addition Pre-blending time Granulation time Impeller and chopper (speed, configuration and location) Spray nozzle type and location Liquid/solid ratio Binder addition method Binder temperature Vessel temperature Fill level	Content, Content uniformity, Granule size, Granule size distribution, Granule hardness, Tapped density, Bulk density, Moisture content, Flow properties, True density
Twin-screw	Screw speed Screw configuration/type/size Liquid/solid ratio Feed rate Fill level Number of die holes	
Fluidized bed	Blending time Spray nozzle (type, quantity, pattern, configuration) Binder addition method Binder fluid temperature Binder addition rate Inlet air (Flow rate, volume, temperature) Filter (properties, size) Shaking intervals Product temperature	

**Table 3:** Summary of engineering-based modeling method applied in wet granulation process.

Equipment Type	Model (Manufacturer)	Scale and Parameter Conditions	Dimensionless Number	Measured QAs	Ref.
High shear/ Low shear	Eirich R02 (EIRICH Machines, Inc., Hardheim, Germany)	Spray nozzle: 0.3, 0.5 and 1.2 mm Amount of binder: 225 g Rotation speed: 1500 rpm	Spray flux	Particle size distribution	[5]
	Cyclomix high-shear granulators (Hosokawa Micron BV, Doetinchem, Nederland)	Vessel size: 1, 5, 10, 250 and 500 L Fill level: 40%	Constant Froude number Constant shear stress Constant impeller tip speed	Strength of granules	[107]
	SPG (Fuji Paudal Co., Ltd., Osaka, Japan)	Vessel size: 2–112 L	Impeller tip speed	Particle flow and collision energy determined from DEM	[28]
	MiPro (ProCepT, Zelzate, Belgium), Fielder PMA 65 (GEA Niro Aeromatic Fielder, Paris, France)	Vessel size: 1.9 and 66 L Fill level: 20 and 40%	Modified dimensionless torque number relevant of Froude number, fill ratio and, impeller clearance at the vessel base	Observation of the surface velocity of the powder bed.	[108]
	TRIAXE system (TriaProcess, Albi, France)	Vessel size: 48 L Gyrational speed of agitator: 25–800 rpm Rotational speed of agitator: 25–800 rpm Fill level: 10 and 20%	Modified Froude and Power numbers	Power consumption	[109]
	Diosna P1–6 (Diosna Dierks und Söhne GmbH, Osnabrück, Germany)	Vessel size: 1.23–100 L Impeller tip speed: 3.8 and 7.5 m/s Fill ratios: 32.2%	Constant impeller tip speed Constant Froude number	Size distribution of the granules	[99]
	Collette Gral (GEA-Colette, Wommelgem, Belgium)	Vessel size: 8, 25, 75 and 600 L	Dimensionless power relationships	Consistency in a commercial mixer torque rheometer	[110]
	MP 20, 90, MPH 200 (GEA Pharma Systems-Collette, Wommelgem, Belgium)	Vessel size: 5–200 L	Dimensionless numbers of Power, Reynolds and Froude number	Power consumption	[111]
	Fielder PMA (GEA Niro Aeromatic Fielder, Paris, France)	Vessel size: 25, 100 and 600 L	Dimensionless numbers of Power, Reynolds and Froude number	Power consumption	[105]
	Fielder PMA (GEA Niro Aeromatic Fielder, Paris, France)	Vessel size: 10, 65, 150 and 300 L Impeller tip speed: about 5.1 and 10.3 m/s Chopper speed: 3000 rpm	Constant impeller tip speed Binder solution proportional to batch size Maintains granulation time proportions about impeller speeds that vary with batch size	Percent loss on drying, granule size distribution, bulk and tap densities	[98]
	Ploughshare Mixer (Gebrüder Lödige Maschinenbau GmbH, Paderborn, Germany)	Vessel size: 40 and 140 L Fill level: 40%	Constant Froude number Constant relative swept volume Constant impeller tip speed	Granule bulk density, sphericity, packing coefficient	[9]
	Collette Gral (GEA-Colette, Wommelgem, Belgium)	Vessel size: 8, 75 and 300 L	Constant Froude number Constant relative swept volume Constant impeller tip speed	Power consumption, temperature of the mass	[100]
	SPG-10, 25, 200, 400 (Fuji Paudal Co., Ltd., Osaka, Japan)	Vessel size: 9.8, 25.7, 205.9 and 401.8 L Binder content: 22% Operating time: 10 min Chopper speed: 3000 rpm	Constant tip speed	Strength, size distribution and compressibility of granules	[112]
Ribbon granulator	S-50 mixer (S. Howes LLC, New York, USA)	Vessel size: 0.5 and 100 ft^3^ Fill level: 40–80% Impeller speeds: 20, 40 and 60 rpm	Adjusting the rotation speed to have the same number of Froude in each vessel size	Content uniformity	[113]
	Helical double-ribbon impeller blender (Custom-made)	Fill level: 8–50% Impeller speeds: 25, 50 and 75 rpm	Correlation between the power number and the cohesion number	Power consumption Content uniformity	[95]
Fluidized bed	NQ-125. 230, 500 (Fuji Paidal Co., Ltd., Osaka, Japan)	Vessel diameter: 125, 230 and 500 mm	The constant ratio of kinetic energy by agitator rotation	Moisture content	[114]
Twin-screw	-	Feed rate: 10, 15 and 20 kg/h Screw speed: 200 and 400 rpm	Dimensionless number about mean residence time and mean time delay	Residence time, mean residence time, fill level	[115]

**Table 4:** Summary of PAT-based modeling method applied in wet granulation process.

Equipment Type	Model (Manufacturer)	PAT Tool (Model)	Detail	Measured QAs	Ref.
High-shear/ Low shear	MiPro (ProCepT, Zelzate, Belgium)	Power consumption	Impeller torque was measuring at bench-scale Current value of the motor at pilot scale	Impeller torque	[108]
		Image analysis (FastCam PCI 1000)	Measuring the powder surface velocity using high speed camera	Particle surface velocity	
	PharmaConnect™ (GEA, Düsseldorf, Germany)	Power consumption (in-line DFF ^2^ sensor)	Measurement range of ± 3 N Measured at 500 point per second with DFF sensor	Impeller torque	[123]
	Fielder PMA (GEA Niro Aeromatic Fielder, Paris, France)	Power consumption (mixer-torque rheometer, Caleva MTR)	Measures impeller torque when rotating at 50 rpm Mix for 30 s, then log data for 30 s	Impeller torque	[8]
	Lodige granulator (Gebrüder Lödige Maschinenbau GmbH, Paderborn, Germany)	FBRM ^1^ and parsum^TM^	Measured in the 790 nm Scanning beam velocity was 4 m/s	Particle size distribution	[44]
	Loedige M5 high-shear mixer (Gebrüder Lödige Maschinenbau GmbH, Paderborn, Germany)	Power consumption (Sineax Type PQ 502), Temperature	Power consumption is calculated by the actual current consumption of the motor	Power consumption Granule temperature	[93]
	Jacketed beken duples mixer (Beken Engineering, London, UK)	Power consumption (Plasti-Coder rotational torque rheometer)	Torque measurement after 3–5 min of mixing	Impeller torque	[124]
	MiPro (ProCepT, Zelzate, Belgium)	NIR ^3^ (Foss Nirsystems Model 6500 Rapid Content Analyzer)	Measured in the range of 1100–2500 nm Spectrum was obtained of 32 scans	Moisture content	[125]
	SPG25 (Fuji Paudal Co., Ltd., Osaka, Japan)	Power consumption (Digital power meter)	Torque of impeller and chopper measured	Particle size distribution Granule moisture content Granule temperature	[40]
		Image analysis (image processing system comprising a CCD ^4^ camera)	Use of high energy Xenon lamps flashing in 1us intervals		
	SPG25, 200 and 400 (Fuji Paudal Co., Ltd., Osaka, Japan)	Power consumption (digital torque meter)	Calculation of agitation power per unit volume using shaft torque.	Shaft torque	[104]
	MiPro (ProCepT, Zelzate, Belgium)	Raman (Renishaw InVia Raman mapping instrument)	Center of the measurement range was settled at 1100 cm^−1^ 45 mW laser source at 785 nm	Distribution of binder within the granules	[38]
	Collette Gral-10 (GEA-Colette, Wommelgem, Belgium),	NIR (Multi-Purpose Analyzer FT-NIR spectrometer),	Laser wavelength was the 785 nm line from a 785 nm	Content uniformity	[126]
		Raman (RamanRxn1 spectrometer)	Measured in the range of 12,500–4000 cm^−1^ with 8 cm^−1^ resolution Spectrum was obtained the average of 16 scans		
Fluidized bed	ConsiGma™ system (GEA-Colette, Wommelgem, Belgium)	FBRM (FBRM M680)	Probe including a scraper unit Scan speed can be adjusted from 2 to 8 m/s	Particle size distribution	[43]
	Glatt GPCG 30/50 (Glatt GmbH, Binzen, Germany)	NIR (NIRSystems 6500 spectrophotometer)	Measured in the range of 1100–2500 nm every 2.5 min	Moisture content	[120]
	FD-3S (Powrex, Hyogo, Japan)	NIR (WET EYE, WETRON)	The 1990 nm wavelength for water detection The 1740 nm wavelength for correction of the background level The 2145 nm wavelength as a non-water-sensitive reference	Moisture content	[127]
	Custom apparatus				
	NQ-160 (Fuji Paidal Co., Ltd., Osaka, Japan)	Image analysis	Use of air purge to prevent visual disturbances due to powder attachment	Particle size distribution, Granule shape	[39]
	GPCG 3 granulator (Glatt GmbH, Binzen, Germany)	Parsum^TM^ (IPP-70 Se SFT-sensor)	The sensor was installed at three different insertion depths The rotation angle of the probe changed in steps of 45 °	Particle size distribution	[128]
	AGM-2A-PJ (Hosokawa Micron Ltd., Osaka, Japan)	Acoustic emissions (AE-901S)	The receptor used had a resonance point of 140 kHz	Particle fluidization	[48]
Twin-screw	ConsiGma-25 Unit (GEA Pharma systems, Collette, Wommelgem, Belgium)	Raman (RamanRxn1 spectrometer)	400 mW laser source at 785 nm. Measured at a resolution of 4 cm^−1^ with an exposure time of 30 s	Solid-state behavior	[129]
		NIR (Fourier-Transform NIR spectrometer)	Measured in the range of 4500–10,000 cm^−1^ with 16 cm^−1^ resolutions. Spectrum was obtained averaged over 16 scans		
		In-line Spatial Filter Velocimetry probe	Semiconductor laser diode probe radiating visible light at a wavelength of 670 nm.	Particle size distribution	
	Thermo Scientific™ Pharma 11 twin-screw granulator (Thermo Fisher Scientific, Karlsruhe, Germany)	Eyecon™	The integration time was 60 s The maximum detection diameter was 3000 µm the analysis block size was 501 pixels	Particle size distribution	[130]
	TS16 Quick Extruder (QUICK 2000 Ltd., Tiszavasvári, Hungary)	Raman (Kaiser RamanRxn2 Hybrid in situ analyzer)	Measured in the range of 200–1890 cm^−1^ with a 4 cm^−1^ resolution Spectrum pixel was 1690	Content uniformity of powder and tablet	[37]

^1^ FBRM: focused beam reflectance measurement; ^2^ DFF: drag flow force; ^3^ NIR: near-infrared spectroscopy; ^4^ CCD: charge-coupled device.

**Table 5:** Summary of physics-based modeling methods applied in the wet granulation process.

Equipment Type	Simulation Tool	Summary	Predicted Attributes	Ref.
High-shear/ Low-shear	DEM	Parameter study of particle shape and impeller geometries Comparison of different vessel sizes (0.173, 1.39 11.1 and 88.7 L)	Blade-bed stress and bed surface velocities	[133]
	PBM–DEM	Wet granulation processes were simulated by the coupled model of PBM and DEM The coupled model demonstrated sensitivity to the impeller speed	Particle size distributions and collision rate functions	[84]
	DEM	Granulator (SPG, Fuji Paudal Co., Ltd.) was used Identification of kinematic and dynamic similarities using DEM Comparison of different vessel sizes (simulated 2, 10, 26, 112 and 206 L)	Internal particle flow Particle collision energy	[28]
	DEM	Comparison kinematic and dynamic similarities of different vessel sizes (1.0, 3.4, 8.1 and 16.0 L)	Particle collision energy (dynamic similarity) Particle velocity (kinematic similarity)	[64]
	DEM	Granulator (Mycromix, Bosch Packaging Technology) was used Confirmation of the effect of the size of the granulator (10 and 600 L) Confirmation of the influence of the number of blades (2 or 3 blades)	Shear forces Force distributions	[6]
	DEM	Granulator (Hosokawa Micron B.V.) was used Analysis of particle breakage and deformation behavior according to vessel size (1, 5 and 50 L)	Velocity fields of granules Stress fields of granules	[107]
	DEM	Identification of parameters affecting granule production using DEM Comparison of different vessel sizes (1, 5, 50 and 250 L)	Velocity fields of granules Stress fields of granules	[136]
	DEM	Effect of mixer size (1–300 L) and fill level (17%, 32% and 46%)	Granule of flow patterns Velocity field and stress of granule bed	[137]
	DEM	Comparative study of particle behavior using PEPT ^1^ and DEM	Internal flow fields and blending patterns	[138]
	DEM	The effects of blade rake angle and blade speed	Velocity fields of particles	[139]
Fluidized bed	CFD–DEM–PBM	CFD–DEM–PBM coupled model for predicting fluidized bed granulation behavior	Average particle size Particle size distribution Moisture content	[85]
	CFD	Predicting the behavior of fluidized bed granulators with different batch sizes (150 g, 2 kg and 45 kg)	Particle volume fraction Bed heights in granulator	[78]
	DEM–CFD	Modified DEM–CFD model using model for particle wetting.	The residence time distribution Average particle velocity and collisions	[140]
	CFD–DEM–PBM	Development and validation of a coupled CFD–DEM–PBM model The effect of process parameters, such as the inlet air flow rate, air temperature and spray rate	Particle velocities, temperature and collision frequencies form DEM Particle residence time from CFD Particle size distribution and moisture content change from PBM	[82]
	DEM–CFD	Particle motion and collision dynamics simulation using the coupled DEM–CFD model Comparison of Top-spray, Wurster-coater and Prismatic shaped spouted bed	Particle velocity Collision velocity between particles Collision velocity between particles and walls Collision frequency	[134]
Twin-screw	DEM-PBM	Evaluate the effect of viscosity and amount of binder, as well as screw speed and type on granules using the coupled DEM-PBM model	Porosity and size distribution, liquid content for PBM Moisture content, porosity, average particle velocity and collision rate for DEM	[141]
	DEM-PBM	Granulator (16 mm Prism EuroLab TSG ^2^, Thermo Fisher Scientific) was used Assess coagulation, breakage and consolidation during granulation using a coupled PBM–DEM model Evaluate the effect of screw configuration on granule quality attributes	Size distribution, liquid distribution and porosity of granules	[83]
	DEM	Granulator (16 mm Prism EuroLab TSG, Thermo Fisher Scientific) was used A study on the granulation performance according to the viscosity of binder solution and the rate of powder feeding	Surface velocities of dry and wet powders Particle size, porosity and strength	[135]

^1^ PEPT: positron emission particle tracking; ^2^ TSG: twin-screw granulator.

**Table 6:** Summary of engineering-based modeling method applied in dry granulation process.

Equipment Model (Manufacturer)	Scale and Parameters Conditions	Dimensionless Number	Measured QAs and Predicted QAs	Ref.
Mini-Pactor (Gerteis Maschinen + Process Engineering AG, Jona, Switzerland)	Roll forces: 3.0–7.5 kN/cm Roll gaps: 1.5, 2.5 and 3.5 mm	Johanson’s model Johanson’s model was modified overestimation of maximum roll surface pressure	Ribbon density Maximum roll surface pressure	[147]
Gerteis (Gerteis, Rapperswil-Jona, Swiss), L.B. Bohle (L.B BOHLE, Ennigerloh, Germany)	Roll width: 25 and 100 mm Specific compaction force: 4, 6 and 8 kN/cm Roll gap: 1.5, 2.25 and 3 mm Roll speed: 2, 3 and 4 rpm	Reynolds model is applied to scale-up the process	Ribbon relative density.	[17]
WP 120 Pharma (Alexanderwerk, Remscheid, Germany)	Roll pressure: 40, 60 and 80 bar Roll speed: 5, 7.5 and 10 rpm Screw speed: 25, 30 and 35 rpm	Johanson’s model	Ribbon porosity	[150]
WP 120 Pharma (Alexanderwerk, Remscheid, Germany)	Roll width: 40 and 100 mm	Modified Bingham number	Ribbon density Ribbon solid fraction	[143]
WP 120 Pharma, WP 200 Pharma (Alexanderwerk, Remscheid, Germany)	Roll pressure: 40, 55 and 70 bar Roll speed: 4, 5, 8 and 12 rpm Screw speed: 19–53 rpm	Modified Johanson’s model	Ribbon density	[14]
Chilsonator IR-220, IR-520 (The Fitzpatrick Co., IL, USA)	Roll pressure: 3–12 kN/cm VFS/HFS ^1^: 4–100 Roll gap: 2–4 mm	Joint-Y partial least squares (JYPLS)	Ribbon density Ribbon solid fraction	[151]
WP 120 Pharma, WP 200 Pharma (Alexanderwerk, Germany)	Roll width: 25, 40 and 75 mm Roll diameter: 120 and 200 mm Roll pressure: 20–121 bar Roll gap: 1.2–4.0 mm	Maintain the ratio between the roller gap and the roller diameter Johanson’s model Constant ratio of screw speed to roller speed	Ribbon density	[152]
Mini-Pactor (Gerteis Maschinen + Process Engineering AG, Jona, Switzerland)	Roller speed: 1, 2 and 3 rpm Roll gap: 2, 3 and 4 mm Specific compaction force: 2, 3.5, 5, 7 and 9	Johanson’s model Reynolds model	Ribbon density	[149]
WP 200 Pharma (Alexanderwerk, Remscheid, Germany)	Minimum gap width: 2 and 4 mm Inlet stress: 100 and 200 kPa Powder-roll friction coefficient: 0.35 and 0.5	A study to modify the Johanson’s model compared to FEM simulation	Maximum roll pressure and ribbon relative density predicted	[18]
WP 120 Pharma (Alexanderwerk, Remscheid, Germany)	Roll speed: 4 and 12 rpm Roll pressure: 20 and 40 bar Screw speed: 20 rpm	Relationships between ribbon porosity, roll speed, roll pressure, screw speed, true density and roll diameter	Ribbon porosity	[153]
Chilsonator IR 220 (The Fitzpatrick Co., IL, USA)	Roll speed: 3 and 9 rpm Roll pressure: 700 and 1000 psi Screw speed: 19 and 21 rpm			
WP 120 Pharma, WP 200 Pharma (Alexanderwerk, Remscheid, Germany)	Roll pressure: 50–70 bar Gap gap: 2.0–2.6 mm Milling speed: 40–80 rpm	Establish relationship between roller compaction parameters and ribbon thickness and density	Granules QAs: flow, bulk density and particle size distribution Ribbon attributes: ribbon density and thickness	[154]

^1^ VFS: vertical screw speed; HFS: horizontal screw speed.

**Table 7:** Summary of PAT-based modeling method applied in dry granulation process.

Equipment (Manufacturer)	PAT Tool (Model)	Detail	Measured QA	Ref.
Laboratory-scale roller compactor (Alexanderwerk, Remscheid, Germany)	CDI non-contact diffuse reflectance spectrometer (SNIR 278, Control Development Inc. South Bend, IN)	Measured in the range of 1305–2205 nm Averaged over 8 scans	Ribbon density	[156]
WP 120 Pharma (Alexanderwerk, Remscheid, Germany)	In-line NIR ^1^ (Multieye)	Measured in the range of 4555–6600 cm^−1^	Ribbon density Particle size Ribbon mechanical strength	[19]
	Off-line NIR (PerkinElmer Spotlight 400 FT-IR)	Measured in the range of 4000–7800 cm^−1^ with a resolution of 2 cm^−1^ Averaged over 8 scans		
	Image analysis (Eyecon particle imager)	Measure particle size by capturing the color of the particle surface by shining red, green and blue LED on the particle		
	Raman (PerkinElmer RamanMicro 300)	250 mW laser source at 785 nm. Measured in the range of 200-3200 cm^−1^ with an integration time of 5 s Averaging two scans		
Die and punch set (Natoli Engineering Co., MO, USA) on a single station Laboratory Press (Carver, Inc., Wabash, Indiana).	NIR (Control Development)	Measured in the range of 1100–2200 cm^−1^ with a resolution of 4.4 nm Averaged over 16 scans	Content uniformity Moisture content Ribbon density Ribbon tensile strength Ribbon Young’s modulus	[157]
Chilsonator IR 220 (The Fitzpatrick Co., IL, USA)	NIR (Control Development)	Measured in the range of 1100–2200 nm with 1 nm intervals	Ribbon density	[155]
Chilsonator IR 220 (The Fitzpatrick Co., IL, USA)	NIR (Control Development)	Used 35 kW tungsten-halogen light source Measured in the range of 1100–2200 nm with a resolution of 4.4 nm.	Moisture content Ribbon density Ribbon tensile strength Ribbon Young’s modulus	[162]
Pharmapaktor L200/30P (Hosokawa Bepex, Leingarten, Germany)	NIR (NIR spectral sensor, MCS 611 NIR 2.2)	Measured in the range of 980–1900 nm with 1 nm resolution Spectra acquisition time of 100 ms.	Content uniformity Ribbon tensile strength Ribbon Young’s modulus, Ribbon density	[163]
WP 120 Pharma (Alexanderwerk, Remscheid, Germany)	NIR-CI ^2^ (Headwall Photonics model 1002A-00371)	Measured in the range of 1100–1700 nm with a resolution of 7 nm	Ribbon porosity distribution Content of API	[158]
WP 120 Pharma (Alexanderwerk, Remscheid, Germany)	NIR (Antaris II FT-NIR analyzer)	Spectrum was obtained of 64 scans Measured in the range of 10,000–4000 cm^−1^ with 8 cm^−1^ resolution	Moisture content	[58]
	Microwave resonance (Sartorius LMA 320PA microwave moisture analyzer)	Operating at 2.5 GHz		
Chilsonator IR-520 (The Fitzpatrick Co., IL, USA)	Microwave resonance (Panametrics, 5077PR)	Pulse repetition frequency was 1 kHz with 100 ns intervals	Young’s modulus Poisson’s ratio Porosity	[159]
	X-ray micro-CT (SkyScan-1172 XRCT)	The spatial resolution was 14.8 μm/pixel Voltage of 50 kV and a currency of 100 μA	Ribbon density	
Mini-Pactor (Gerteis Maschinen + Process Engineering AG, Jona, Switzerland)	X-ray micro-CT (CT alpha)	Scanned at a resolution of 80 mm per voxel All scans were performed in 1600 projections Voltage of 80 kV and a currency of 80 mA	Ribbon porosity	[164]
laboratory-scale instrumented roller compactor (developed at the University of Birmingham)	X-ray (X-ray micro-CT system)	Voltage and current were 50 kV and 98 μA The total sample rotation was set at 180° with an interval of 0.9 ° The spatial resolution is 11 μm/pixel	Ribbon density distribution	[160]
Mini-Pactor (Gerteis Maschinen + Process Engineering AG, Jona, Switzerland)	thermographic camera (optris PI 640, optris GmbH, D)	Monitoring frequency was 32 frames per second Distance between ribbon and the camera lens was 10–20 cm	Ribbon density distribution	[161]

^1^ NIR: near-infrared spectroscopy; ^2^ NIR-CI: near-infrared chemical imaging.

**Table 8:** Summary of physics-based modeling method applied in dry granulation process.

Equipment Model (Manufacturer)	Simulation	Summary	Predicted Attributes	Ref.
WP 200 Pharma (Alexanderwerk, Remscheid, Germany)	FEM	FEM simulation was evaluated in comparison with the Johanson’s model	Normal stress Maximum ribbon relative density	[165]
Komarek B050PH laboratory press (K.R. Komarek Inc., IL, USA)	FEM	Comparative study of density distribution of ribbon by FEM and light transmission	Principal stress and density across the width of the strip.	[167]
Gerteis roll compactor: Mini-pactor 250/25 (Gerteis, Rapperswil-Jona, Swiss)	FEM	Investigate the effect of sealing system design on the density distribution of ribbon using FEM	Ribbon density distribution Roll pressure	[79]
Komarek B050H Laboratory Press (K.R. Komarek Inc., IL, USA)	DEM-FEM	Study on the effect of screw feed rate using DEM-FEM coupling approach	Roll pressure Ribbon relative density	[23]
WP 120 Pharma (Alexanderwerk, Remscheid, Germany)	FEM	Comparison of the results of ribbon density with experimental measurements of FEM simulations	Ribbon density	[166]
RC100 (Roland Research Devices, Inc., NJ, USA)	FEM	Investigate ribbon characteristics according to various process parameters with FEM	Roll pressure Ribbon density Feed stress Roll friction on roll force	[168]
WP 200 Pharma (Alexanderwerk, Remscheid, Germany)	FEM	Improvement of prediction accuracy of predicted ribbon density at Johanson’s roll compaction model using FEM	Roll pressure Ribbon relative density	[18]
Komarek B050PH laboratory press (K.R. Komarek Inc., IL, USA)	FEM	Study the mechanism of powder transport and ribbon density by predicting the pressure distribution between particles and roller	Roll pressure distribution, shear stress and nip angle	[169]
